# Design, Synthesis, Anticholinesterase and Antidiabetic Inhibitory Activities, and Molecular Docking of Novel Fluorinated Sulfonyl Hydrazones

**DOI:** 10.1021/acsomega.4c07160

**Published:** 2024-09-25

**Authors:** Bedriye Seda Kurşun Aktar

**Affiliations:** Department of Hair Care and Beauty Services, Yeşilyurt Vocational School, Malatya Turgut Özal University, Malatya 44210, Turkey

## Abstract

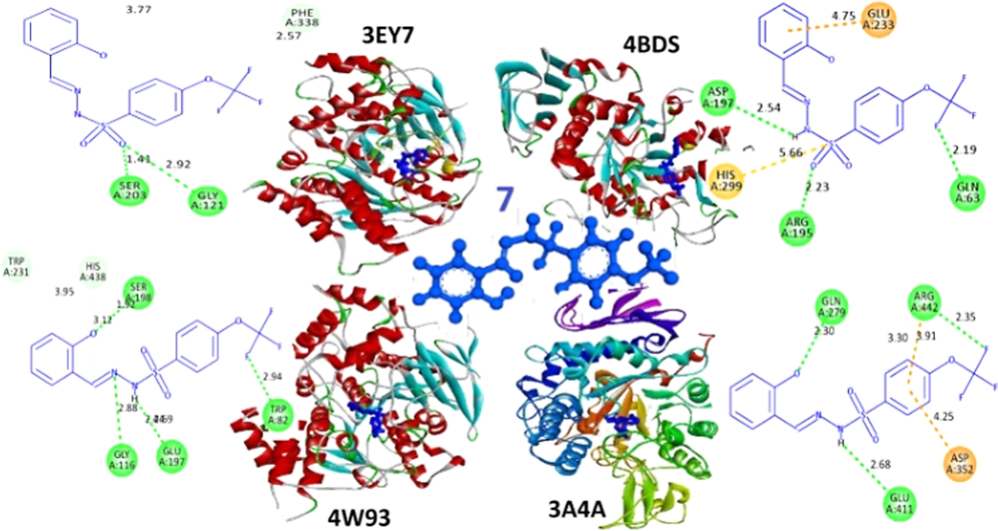

In this study, it was aimed to synthesize (*E*)-*N*′-(2-hydroxybenzylidene)-substituted benzenesulfonohydrazide (**1**–**7**) from the 2-hydroxybenzaldehyde reaction of different substituted fluorinated sulfonyl hydrazides. The structures of the synthesized molecules were characterized by elemental analysis, FTIR, ^1^H NMR, ^13^C NMR, ^19^F NMR, and 2D NMR (HMBC, correlation spectroscopy, and HQSC). The anticholinesterase (AChE and BChE) and antidiabetic (α-glucosidase, α-amylase) inhibition activities of the synthesized compounds were evaluated. According to biological activity test results, (*E*)-*N*′-(2-hydroxybenzylidene)-4-(trifluoromethoxy)benzenesulfonohydrazide (compound **7** among hydrazone derivatives **1**–**7**) demonstrated better BChE inhibitor activity than galantamine in anticholinesterase inhibition; and in the α-glucosidase and α-amylase assay, it exhibited more antidiabetic inhibition activity than the reference standard.

## 1 Introduction

**Figure 1:**
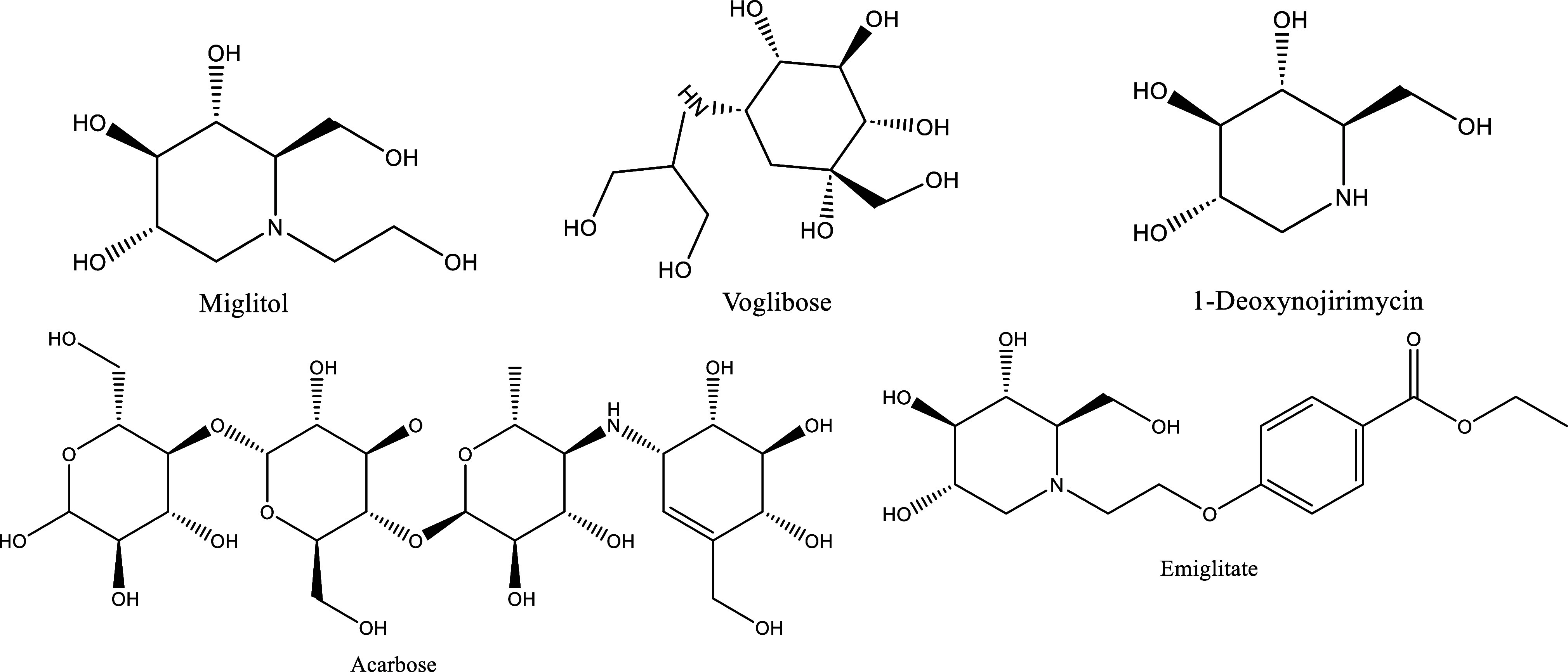
Chemical structures of some α-glucosidase and α-amylase inhibitors.

**Figure 2:**
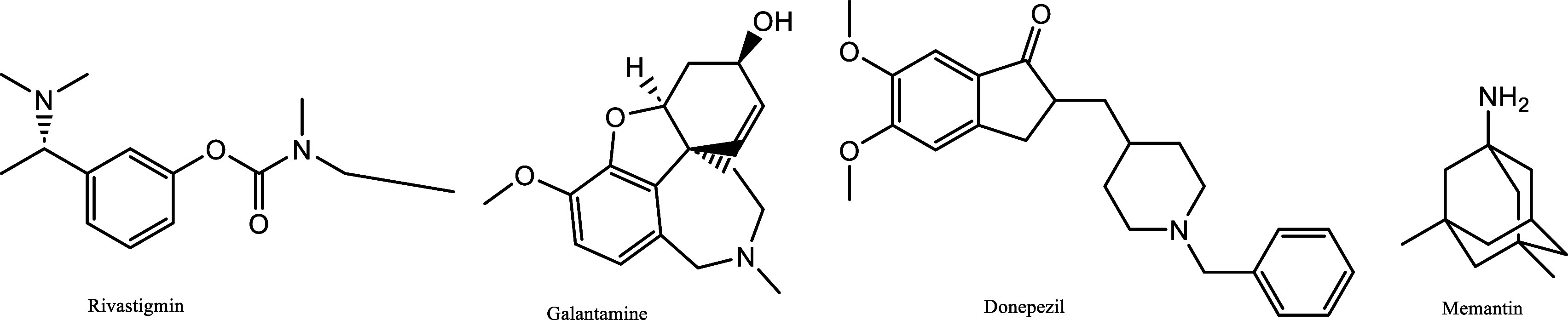
Some FDA-approved drugs for Alzheimer’s disease (AD).

The number of patients with Alzheimer’s disease (AD) and diabetes mellitus (DM) is increasing at an alarmingly rapid rate. This increase places a great burden on both the country’s health system and its economy. It is estimated that the number of patients with diabetes will reach 643 million and the number of patients with Alzheimer’s will reach 74.7 million in 2030.^1,2^ Diabetes mellitus is a lifelong metabolic disease that develops when the gland called the pancreas does not produce enough insulin hormone or the insulin hormone it produces is not used effectively. Diabetes mellitus is a global public health problem that is becoming increasingly more common all over the world and has a high risk of death.^3^ Population growth, aging, urbanization, physical inactivity, obesity, and stress can be given as examples of the main risk factors that increase the prevalence of DM.^4^ In 2021, there were 537 million adult patients between the ages of 20 and 79. It is estimated that this number will increase to 643 million in 2030 and 783 million in 2045.^5^ Diabetes mellitus affects carbohydrate, protein, and lipid metabolisms in the long term; it causes various organ failures such as those of the eyes, heart, and kidneys, as well as nerves and blood vessels to fail to function properly.^6,7^ The α-amylase and α-glucosidase inhibitors on the market are acarbose, 1-deoxynojirimycin, voglibose, and miglitol (Figure 1) (see Figure 2).

Alzheimer’s disease is not an irreversible and completely treatable disease. The medications used in treatment only aim at slowing down the process and reducing the severity of symptoms. In this disease, even slowing down the process is of great importance as the course of the disease increases day by day and the patient will have to live dependent on others for their daily life. In Alzheimer’s disease, drug design is critical in order to find drugs that aim to prevent the formation and precipitation of certain proteins that damage the functions of the brain and nerve cells so that the patient can meet the needs of daily life.^8^ There are five drugs approved by the FDA (the U.S. Food and Drug Administration) for the treatment of this disease. These are drugs called tacrine, donepezil, rivastigmine, galantamine, and memantine. Among these, tacrine is not used in treatment due to the high number of side effects.

In studies conducted in recent years, scientists named Alzheimer’s disease (AD) “Type-3 diabetes” due to the common molecular and cellular features between memory impairments, cognitive decline, and insulin resistance in elderly individuals. There is a strong but elusive relationship between type 2 diabetes mellitus and Alzheimer’s. Both are linked to insulin resistance, insulin growth factor (IGF) signaling, glycogen synthase kinase 3β signaling mechanism, inflammatory response, oxidative stress, neurofibrillary tangle formation, amyloid beta (Aβ) formation, and regulation of acetylcholine esterase activity. There are common mechanisms between diabetes mellitus (Type-1 and Type 2) and Alzheimer’s disease (AD). Therefore, for both diseases, there is a need for treatments that can perform multiple tasks by inhibiting drug targets. For this reason, both antidiabetic and anti-Alzheimer’s activities were examined in this study.^9−11^

**Figure 3:**
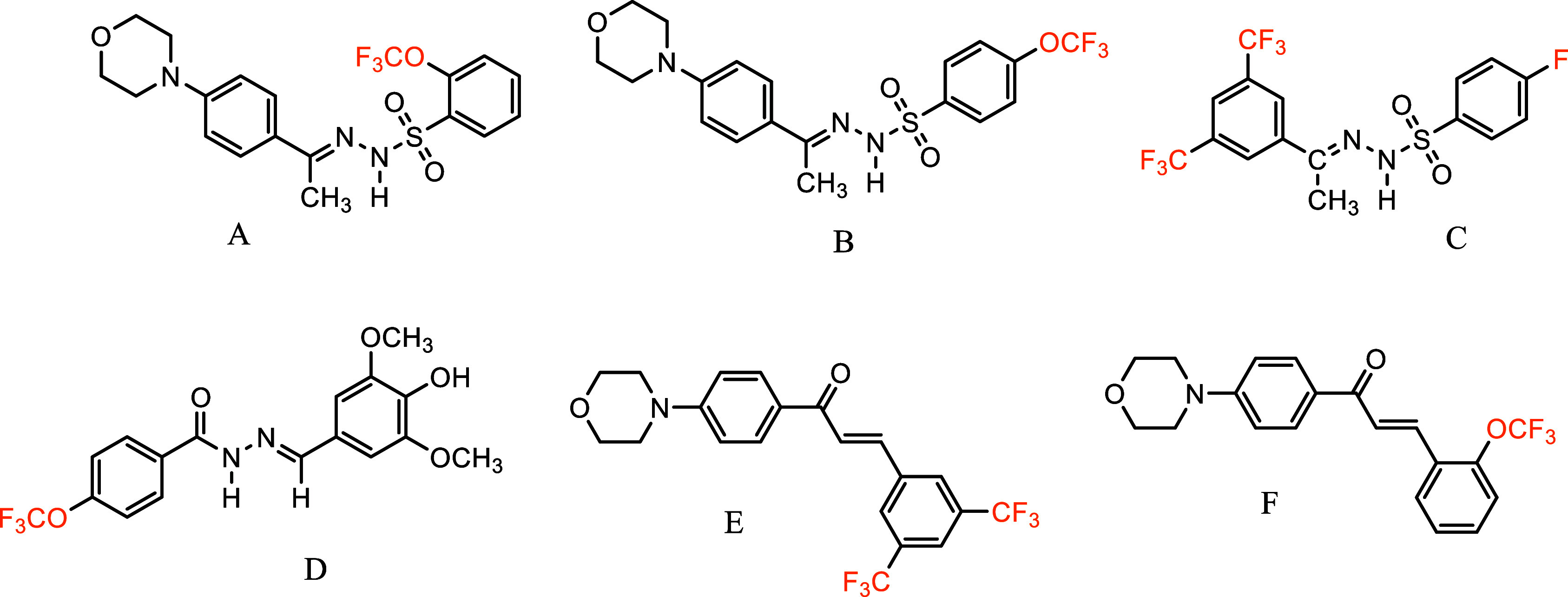
Drawings of active molecules of fluorinated compounds.

In our previous studies, molecules with F, CF_3_, and OCF_3_ groups attached to the phenyl ring at different positions were found to have BChE inhibitory activity (A),^12^ antioxidant activity (B),^12^ tyrosinase inhibitory activity (C),^13^ and β-carotene-linoleic acid and ABTS cation radical scavenging activities (D).^14^ Furthermore, the antiproliferative activity [Hela cell line (E) and C6 cell line (F)] was found to be high (Figure 3).^15^

**Figure 4:**
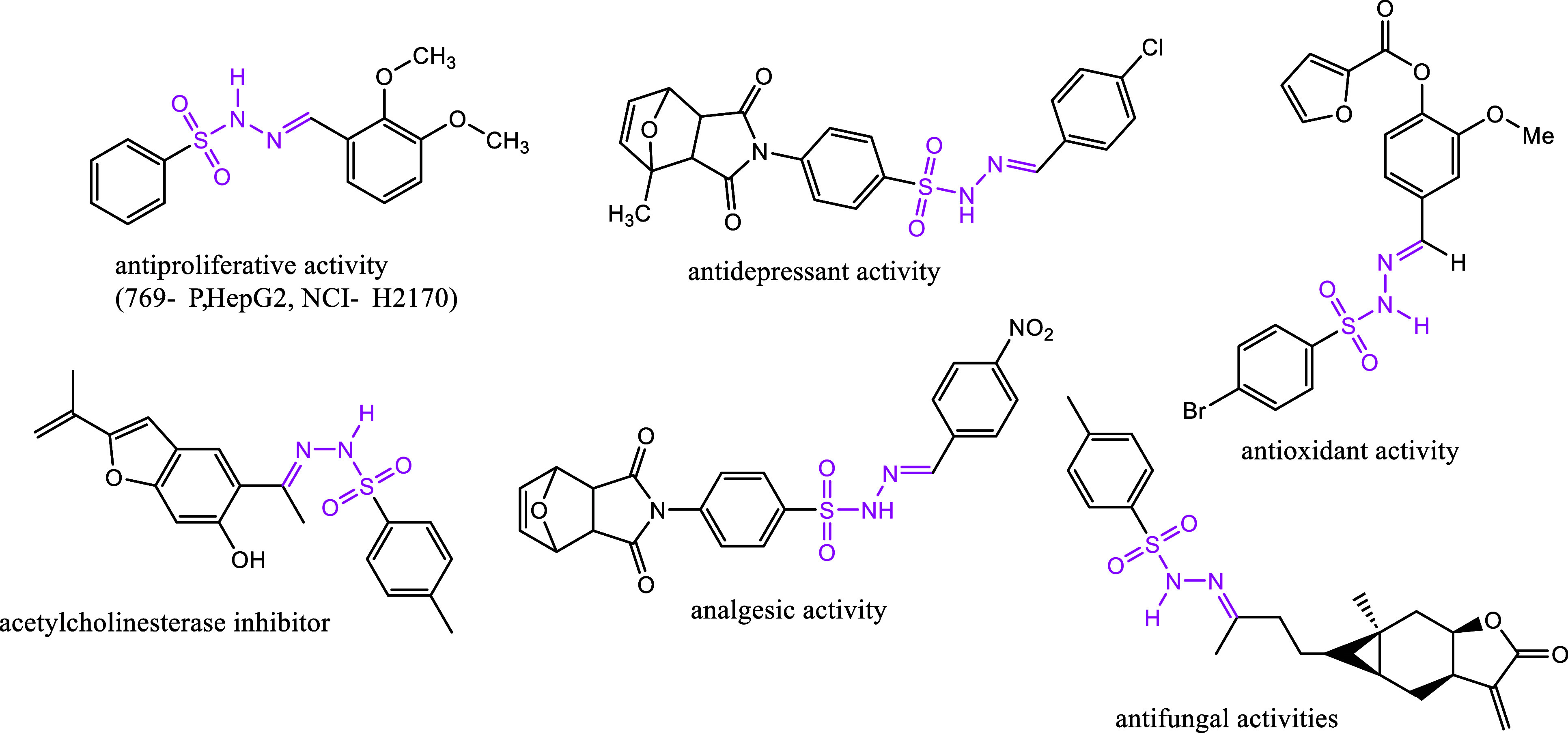
Drawings of active molecules of sulfonyl hydrazones.

While designing molecules in this study, sulfonyl hydrazones were synthesized by selecting fluorinated sulfonyl chlorides due to their binding selectivity, metabolic stability, and lipophilic properties of fluorinated compounds. Sulfonyl hydrazones have an important place in pharmaceutical chemistry due to their structures. Hydrazones show pharmacophore properties thanks to the nitrogen atoms in their structure. The sulfone group in the sulfonyl hydrazone is a group that increases the solubility and is used frequently. Sulfonyl hydrazones (SO_2_NHN=CH−) are of great importance in drug designs because they are rich in biological activity. Sulfonyl hydrazones have analgesic,^16^ antifungal,^17^ anti-Alzheimer’s,^12,13^ acetylcholinesterase,^18^ antidepressant,^19^ anticancer,^20^ antibacterial,^21^ and antioxidant^22^ activities (Figure 4).

## 2 Experimental Section

### 2.1 Materials and Methods

Chemicals and solvents were of analytical grade and purchased from Merck, Apollo, and Sigma-Aldrich. All chemical reactions were monitored with thin layer chromatography (TLC) using Merck silica gel 60 F254 plates. The melting point of 18 was taken automatically with a Stuart SMP20 instrument. FTIR spectra were determined with a PerkinElmer 1620 model FTIR spectrophotometer. Elemental analyses (CHNS) were performed on a VarioMICRO elemental analyzer. ^1^H NMR, ^13^C NMR, ^19^F NMR, correlation spectroscopy (COSY), HMBC, and HQSC spectra were recorded on an Agilent 600 and 400 MHz spectrometer. All biological activity measurements were done using a 96-well microplate reader (SpectraMax 340PC384, Molecular Devices, USA).

### 2.2 Enzyme Inhibition Activities

#### 2.2.1 Determination of Anticholinesterase Activity of Compounds (**1**–**7**)

The electric eel acetylcholinesterase (AChE, Type–VI-S, EC 3.1.1.7, 425.84 U/mg) and horse reddish butyrylcholinesterase (BChE, EC 3.1.1.8, 11.4 U/mg) were used to determine the anticholinesterase activity. Compounds **1**–**7** were where acetylthiocholine iodide and butyryl-thiocholine chloride were employed as substrates using the spectroscopic method.^23^ In brief, 130 μL of sodium phosphate buffer (100 mM, pH 8.0), 10 μL of compounds **1**–**7** in DMSO at different concentrations, and 20 μL of AChE or BChE buffer were mixed. After incubation for 15 min at 25 °C, 20 μL 0.5 mM DTNB (5,50-dithiobis(2-nitrobenzoic acid)) and 20 μL acetylthiocholine iodide (0.71 mM) or butyryl-thiocholine chloride (0.2 mM) were added. Then, the absorbance was measured at 412 nm.

A kinetic study was performed to find out how the compound showing the inhibitory effect binds to the enzyme and its inhibition constant. Lineweaver–Burk plots were drawn from the data obtained as a result of the experimental study.^24^

### 2.3 Determination of α-Amylase Inhibitory Activity of Compounds **1**–**7**

The α-amylase inhibitory activity of compounds **1**–**7** was tested by using the spectroscopic method with slight changes.^25^ In brief, 25 μL of sample solution in different concentrations was mixed with 50 μL of α-amylase solution (0.1 U/mL) in phosphate buffer (20 mM pH 6.9 phosphate buffer prepared with 6 mM NaCl) in a 96-well microplate. The mixture was preincubated for 10 min at 37 °C. After preincubation, 50 μL of starch solution (0.05%) was added and incubated for 10 more min at 37 °C. The reaction was stopped by adding 25 μL of HCl (0.1 M), and then 100 μL of Lugol solutions was added for monitoring. A 96-well microplate reader was used to measure absorbance at 565 nm.

### 2.4 Determination of α-Glucosidase Inhibitory Activity of Compounds **1**–**7**

The α-glucosidase inhibitory activity of compounds **1**–**7** was determined using the spectroscopic method with slight modifications.^26^ In brief, 50 μL of phosphate buffer (10 mM pH 6.9), 25 μL of PNPG (*p*-nitrophenyl-α-d-glucopyranoside) in phosphate buffer (10 mM pH 6.9), 10 μL of sample solution, and 25 μL of α-glucosidase (0.1 U/mL) in phosphate buffer (10 mM pH 6.0) were mixed in a 96-well microplate. After 20 min of incubation at 37 °C, 90 μL of Na_2_CO_3_ (100 mM) was added into each well to stop the enzymatic reaction. Absorbance of the 96-well microplate reader was recorded at 400 nm.

### 2.5 In Silico Studies

#### 2.5.1 Molecular Docking Studies

A molecular docking study was carried out in order to understand the details of the possible interactions of the synthesized molecules with enzymes at the molecular level. 3D SDF structures of the molecules were prepared using the MarvinSketch 23.17 program. The crystal structures of the enzymes for the docking studies were downloaded from the Protein Data Bank (http://www.rcsb.org) with identification codes as follows: α-glucosidase (PDB code: 3A4A), α-amylase (PDB code: 4W93), AChE (PDB code: 4EY7), and BChE (PDB code: 4BDS).^27−30^ The structural refinement of proteins was conducted using Molegro Virtual Docker software, where careful examination and correction of structural errors were undertaken.^31^ The selection of docking regions was guided by the cavities identified in the crystal structure where the reference ligand binds. To validate the efficacy of the docking protocols employed, a redocking procedure was executed using the cocrystallized ligand. Ten docking experiments were performed for the synthesized molecules in the active sites of the enzymes. Subsequently, molecular interactions between enzymes and compounds exhibiting a higher binding affinity were visualized using Discovery Studio Visualizer 2020 software.

### 2.6 In Silico Pharmacokinetic Analyses

Fluorinated sulfonyl hydrazones; absorption, distribution, metabolism, and excretion (ADME) properties; and basic parameters affecting drug metabolism (molecular weight, H-bond acceptor, H-bond donor, TPSA, Lipinski, iLogP, GI absorption, and BBB permeability) were determined using the web-based SwissADME program.^32^ Computational toxicity risk parameters (reproductive, irritant, mutagenic, and tumorigenic effects) of compounds **1**–**7** were calculated in the Organic Chemistry Portal program.^33^

### 2.7 Analysis of Physical and Spectroscopic Data of Synthesized Compounds (**1**–**7**)

#### 2.7.1 Synthesis of (*E*)-*N*′-(2-Hydroxybenzylidene)-Substituted Benzenesulfonohydrazide (**1**–**7**)

A mixture of 2-hydroxyaldehyde (0.01 mol), different substituted fluorinated sulfonyl hydrazides (0.01 mol), and a few drops of glacial acid were refluxed in acetonitrile for 6 h. A few drops of glacial acid were added to the reaction. When the reaction was complete, the mixture was cooled, the precipitated solid was filtered, and the precipitate was recrystallized from ethanol.^13^

##### 2.7.1.1 (*E*)-4-Fluoro-*N*′-(2-hydroxybenzylidene)benzenesulfonohydrazide (**1**)

White solid. Yield: 56%, 143–144 mp °C. FTIR ν_max_ (cm^–1^): 3152 (N–H stretching band); 1585 (C=N stretching band); 1169 (SO_2_ symmetrical), 1336 (SO_2_ asymmetric). ^**1**^**H NMR (400 MHz) (DMSO-*d***_**6**_**/TMS): δ ppm**: 6.84 (1H, t, *J*_1_ = 7.60, *J*_2_ = 7.20 Hz), 6.86 (1H, d, *J* = 8.00 Hz), 7.24 (1H, t, *J*_1_ = 6.80, *J*_2_ = 8.80 Hz), 7.48 (2H, t, *J*_1_ = 8.80, *J*_2_ = 6.80 Hz), 7.49 (1H, d, *J* = 8.80 Hz), 7.92–7.96 (m, 2H), 8.21 (s, 1H), 10.16 (s, 1H), 11.53 (s, 1H). ^**13**^**C NMR (100 MHz) (DMSO-*d***_**6**_**/TMS): δ ppm:** 116.68, 117.16, 119.56, 119.92, 127.69, 130.67, 132.04, 146.75, 156.97, 163.76, 166.26. **Elemental Analysis:** C_13_H_11_FN_2_O_3_S (g/mol) Anal. Calcd (%): C, 53.06; H, 3.77; N, 9.52; S, 10.89. Found (%): C, 53.26; H, 3.80; N, 9.61; S, 10.97.

##### 2.7.1.2 (*E*)-*N*′-(2-Hydroxybenzylidene)-2-(trifluoromethyl)benzenesulfonohydrazide (**2**)

Cream solid. Yield: 28%, 162–163 mp °C. FTIR ν_max_ (cm^–1^): 3225 (N–H stretching band); 1590 (C=N stretching band); 1178 (SO_2_ symmetrical), 1375 (SO_2_ asymmetric). ^**1**^**H NMR (600 MHz) (DMSO-*d***_**6**_**/TMS): δ ppm**: 6.77 (1H, t, *J*_1_ = 7.20, *J*_2_ = 7.80 Hz), 6.82 (1H, d, *J* = 7.20 Hz), 7.19 (1H, t, *J*_1_ = 7.20, *J*_2_ = 6.60 Hz), 7.40 (1H, d, *J* = 9.60 Hz), 7.83 (1H, t, *J*_1_ = 7.80, *J*_2_ = 7.80 Hz), 7.90 (1H, t, *J*_1_ = 7.80, *J*_2_ = 7.80 Hz), 7.98 (1H, d, *J* = 7.80 Hz), 8.10 (1H, d, *J* = 6.60 Hz), 8.29 (s, 1H), 10.08 (s, 1H), 12.01 (s, 1H). ^**13**^**C NMR (150 MHz) (DMSO-*d***_**6**_**/TMS): δ ppm:** 116.63, 119.59, 119.85, 122.27, 124.09, 127.42, 128.87, 131.61, 131.99, 133.80, 134.00, 138.35, 145.87, 156.88. **Elemental Analysis**: C_14_H_11_F_3_N_2_O_3_S (g/mol) Anal. Calcd (%): C, 48.84; H, 3.22; N, 8.14; S, 9.31. Found (%): C, 48.93; H, 3.36; N, 8.21; S, 9.43.

##### 2.7.1.3 (*E*)-*N*′-(2-Hydroxybenzylidene)-3-(trifluoromethyl)benzenesulfonohydrazide (**3**)

Matter solid. Yield: % 21, 160–161 mp °C. FTIR ν_max_ (cm^–1^): 3225 (N–H stretching band); 1576 (C=N stretching band); 1177 (SO_2_ symmetrical), 1375 (SO_2_ asymmetric). ^**1**^**H NMR (600 MHz) (DMSO-*d***_**6**_**/TMS): δ ppm**: 6.79 (1H, t, *J*_1_ = 9.00, *J*_2_ = 6.00 Hz), 6.82 (1H, d, *J* = 9.60 Hz), 7.20 (1H, t, *J*_1_ = 10.20, *J*_2_ = 5.40 Hz), 7.45 (1H, d, *J* = 5.40 Hz), 7.87 (1H, t, *J*_1_ = 7.80, *J*_2_ = 7.80 Hz), 8.05 (1H, d, *J* = 9.00 Hz), 8.09 (s, 1H), 8.15 (1H, d, *J* = 8.40 Hz), 8.18 (s, 1H), 10.10 (s, 1H), 11.61 (s, 1H). ^**13**^**C NMR (150 MHz) (DMSO-*d***_**6**_**/TMS): δ ppm:** 116.67, 117.46, 119.52, 119.85, 124.14, 127.18, 130.19, 130.41, 131.50, 131.67, 132.17, 140.27, 146.92, 156.97. C_14_H_11_F_3_N_2_O_3_S (g/mol) Anal. Calcd (%): C, 48.84; H, 3.22; N, 8.14; S, 9.31. Found (%): C, 48.94; H, 3.37; N, 8.23; S, 9.42.

##### 2.7.1.4 (*E*)-*N*′-(2-Hydroxybenzylidene)-4-(trifluoromethyl)benzenesulfonohydrazide (**4**)

Cream solid. Yield: 43%, 159–160 mp °C. FTIR ν_max_ (cm^–1^): 3200 (N–H stretching band); 1576 (C=N stretching band); 1161 (SO_2_ symmetrical), 1368 (SO_2_ asymmetric). ^**1**^**H NMR (600 MHz) (DMSO-*d***_**6**_**/TMS): δ ppm**: 6.83 (1H, t, *J*_1_ = 11.40, *J*_2_ = 14.40 Hz), 6.86 (1H, d, *J* = 12.60 Hz), 7.24 (1H, t, *J*_1_ = 12.60, *J*_2_ = 10.80 Hz), 7.50 (1H, d, *J* = 11.40 Hz), 8.03 (2H, d, *J* = 12.60 Hz), 8.09 (2H, d, *J* = 12.60 Hz), 8.23 (s, 1H), 10.15 (s, 1H), 11.76 (s, 1H). ^**13**^**C NMR (150 MHz) (DMSO-*d***_**6**_**/TMS): δ ppm:** 116.68, 119.58, 119.91, 125.21, 127.04, 127.43, 128.63, 132.14, 133.09, 143.16, 146.81, 156.99. C_14_H_11_F_3_N_2_O_3_S (g/mol) Anal. Calcd (%): C, 48.84; H, 3.22; N, 8.14; S, 9.31. Found (%): C, 48.89; H, 3.25; N, 8.17; S, 9.45.

##### 2.7.1.5 (*E*)-*N*′-(2-Hydroxybenzylidene)-2-(trifluoromethoxy)benzenesulfonohydrazide (**5**)

White solid. Yield: 20%, 138–139 mp °C. FTIR ν_max_ (cm^–1^): 3220 (N–H stretching band); 1591 (C=N stretching band); 1160 (SO_2_ symmetrical), 1372 (SO_2_ asymmetric). ^**1**^**H NMR (600 MHz) (DMSO-*d***_**6**_**/TMS): δ ppm**: 6.77 (1H, t, *J*_1_ = 7.80, *J*_2_ = 7.20 Hz), 6.81 (1H, d, *J* = 7.80 Hz), 7.19 (1H, t, *J*_1_ = 8.40, *J*_2_ = 5.40 Hz), 7.36 (1H, d, *J* = 7.80 Hz), 7.57–7.60 (m, 2H), 8.25 (s, 1H), 10.10 (s, 1H), 11.93 (s, 1H). ^**13**^**C NMR (150 MHz) (DMSO-*d***_**6**_**/TMS): δ ppm:** 116.63, 119.50, 119.81, 121.87, 127.62, 128.21, 131.62, 131.94, 136.08, 145.52, 146.06, 156.91. C_14_H_11_F_3_N_2_O_4_S (g/mol) Anal. Calcd (%): C, 46.67; H, 3.08; N, 7.78; S, 8.90. Found (%): C, 46.72; H, 3.13; N, 7.84; S, 8.96.

##### 2.7.1.6 (*E*)-*N*′-(2-Hydroxybenzylidene)-3-(trifluoromethoxy)benzenesulfonohydrazide (**6**)

Yellow solid. Yield: 30%, 137–138 mp °C. FTIR ν_max_ (cm^–1^): 3193 (N–H stretching band); 1580 (C=N stretching band); 1160 (SO_2_ symmetrical), 1336 (SO_2_ asymmetric). ^**1**^**H NMR (600 MHz) (DMSO-*d***_**6**_**/TMS): δ ppm**: 6.82 (1H, t, *J*_1_ = 7.20, *J*_2_ = 8.00 Hz), 6.86 (1H, d, *J* = 8.00 Hz), 7.24 (1H, t, *J*_1_ = 6.80, *J*_2_ = 6.80 Hz), 7.49 (1H, d, *J* = 6.00 Hz), 7.73 (1H, d, *J* = 8.40 Hz), 7.77 (s, 1H), 7.80 (1H, t, *J*_1_ = 7.60, *J*_2_ = 8.00 Hz), 7.91 (1H, d, *J* = 8.00 Hz), 8.21 (s, 1H), 10.15 (s, 1H), 11.67 (s, 1H). ^**13**^**C NMR (150 MHz) (DMSO-*d***_**6**_**/TMS): δ ppm:** 116.66, 119.56, 119.85, 119.92, 126.44, 126.73, 127.16, 132.18, 132.42, 141.16, 146.68, 148.64, 148.66, 156.96. ^**19**^**F NMR:** −57.06. **COSY NMR:** 6.82–7.49, 7.24–6.86, 7.24–7.49. **HSQC NMR:** 6.86–116.70, 6.82–119.84, 7.24–132.36, 7.49–127.35, 7.77–119.71, 7.73–126.61, 7.80–132.58, 7.91–126.73, 8.21–146.89. C_14_H_11_F_3_N_2_O_4_S (g/mol) Anal. Calcd (%): C, 46.67; H, 3.08; N, 7.78; S, 8.90. Found (%): C, 46.75; H, 3.11; N, 7.87; S, 8.97.

##### 2.7.1.7 (*E*)-*N*′-(2-Hydroxybenzylidene)-4-(trifluoromethoxy)benzenesulfonohydrazide (**7**)

White solid. Yield: 36%, 191–192 mp °C. FTIR ν_max_ (cm^–1^): 3152 (N–H stretching band); 1585 (C=N stretching band); 1169 (SO_2_ symmetrical), 1336 (SO_2_ asymmetric). ^**1**^**H NMR (600 MHz) (DMSO-*d***_**6**_**/TMS): δ ppm**: 6.93 (1H, t, *J*_1_ = 9.00, *J*_2_ = 8.40 Hz), 6.95 (1H, d, *J* = 8.40 Hz), 7.32 (1H, t, *J*_1_ = 7.20, *J*_2_ = 9.60 Hz), 7.54 (m, 3H), 8.08 (2H, d, *J* = 9.00 Hz), 8.66 (s, 1H), 11.21 (s, 1H), 12.20 (s, 1H). ^**13**^**C NMR (150 MHz) (DMSO-*d***_**6**_**/TMS): δ ppm:** 116.90, 119.14, 119.86, 121.32, 129.87, 130.53, 132.00, 132.44, 148.97, 151.19, 157.93, 162.14. C_14_H_11_F_3_N_2_O_4_S (g/mol) Anal. Calcd (%): C, 46.67; H, 3.08; N, 7.78; S, 8.90. Found (%): C, 46.74; H, 3.17; N, 7.89; S, 8.99.

## 3 Results and Discussion

It has been reported in the literature that diabetic patients are more likely to have Alzheimer’s disease.^11^ Reducing the complications of diabetes may reduce the progression and occurrence of Alzheimer’s disease.^34^ Therefore, a substance that affects all of the targets used to treat these diseases could be a multifunctional drug. This study is the first to investigate the in vitro anticholinesterase and antidiabetic activities of the synthesized compounds **1**–**7**. Table 1 shows the synthetic pathway and substituent groups (**1**–**7**) carried out (Table 1).

**Table 1:**
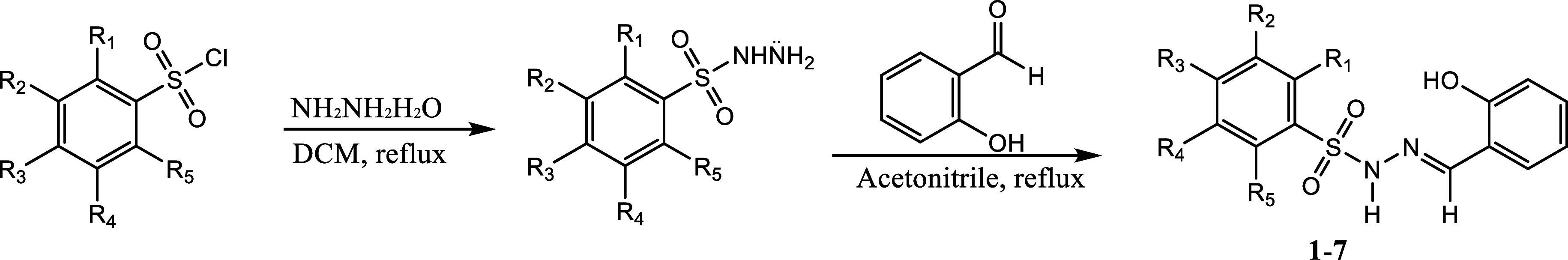
Synthetic Pathway of (*E*)-*N*′-(2-Hydroxybenzylidene)-Substituted Benzenesulfonohydrazide Derivatives (**1–7**)

**compound**	**R**_**1**_	**R**_**2**_	**R**_**3**_	**R**_**4**_	**R**_**5**_
**1**	H	H	**F**	H	H
**2**	**CF**_**3**_	H	H	H	H
**3**	H	**CF**_**3**_	H	H	H
**4**	H	H	**CF**_**3**_	H	H
**5**	**OCF**_**3**_	H	H	H	H
**6**	H	**OCF**_**3**_	H	H	H
**7**	H	H	**OCF**_**3**_	H	H

When the FTIR spectra of the synthesized compounds (**1**–**7**) were examined, it was determined that the C=N bond, which is one of the most determining bands of the hydrazone structure, was stretched in the range of 1576–1591, while the N–H bond was stretched in the range of 3152–3225. Asymmetric and symmetric stretching bands between S=O were detected as 1336–1375 and 1160–1178, respectively. It was determined that the absorption bands of the structures in the FTIR spectra of the synthesized sulfonyl hydrazone compounds (**1**–**7**) were within the ranges specified in the literature.^13,35−39^

When the ^1^H NMR spectra of the synthesized compounds are examined, the biggest evidence of the formation of hydrazone compounds is that the proton peaks at 4–5 ppm that may occur in hydrazide molecules are not observed, but the NH peaks in the C=N–N**H**S=O_2_ skeleton group of the hydrazone structure are observed in the range of 11.53–12:20 ppm. Protons belonging to the O**H** group of compounds **1**–**7** are at 10.08–11.21 ppm; protons of the aldehyde are at 8.18–8.66 ppm; and protons of the aromatic ring were found to resonate at 6.77–8.15 ppm.^13,35−39^

Azomethine (**C**=N) carbon, the most important peak in the ^13^C NMR spectrum, resonates in the 156.88–157.93 ppm range. This peak is one of the most specific peaks of the hydrazone compounds. Aromatic carbon atoms also resonate.^13,35−39^

When ^19^F NMR is examined, since the F atom of OCF_3_ that belongs to compound **6** has a high electronegativity, it was determined that there was a resonance at −57.06 (Figure S19 in Supporting Information).

Further examination of the HSQC, COSY, and HMBC spectra of compound **6** enabled the determination of where the protons and carbons resonate. The HSQC spectrum made it possible to determine which proton and carbon belonged to compound **6**. The three extended spin systems (6.82–7.49, 7.24–6.86, and 7.24–7.49) were determined in the ^1^H,^1^ H correlation spectra (COSY) of compound **6** (Figure S20 in Supporting Information). When HSQC was examined, the matches of 6.86–116.70, 6.81–119.84, 7.24–132.36, 7.48–127.35, 7.77–119.71, 7.73–126.61, 7.80–132.58, 7.90–126.73, and 8.22–146.89 were determined (Figure S21 in Supporting Information). The locations of H and C were precisely determined with the HMBC spectrum (Figure S22 in Supporting Information).^13,35−39^

## 4 Pharmacology

One of the aims of this study was to analyze the anticholinesterase (AChE and BChE) and antidiabetic (α-glucosidase and α-amylase) inhibition activities of the synthesized compounds.

### 4.1 Anticholinesterase Inhibition Activity

The anticholinesterase inhibition activity of the synthesized hydrazone derivatives (**1**–**7**) was tested against AChE and BChE. Anticholinesterase inhibition activity results are given in Table 2. Within the synthesis series, compound **7** was determined to be the most active compound in both AChE and BChE, and it was more active than the positive standard galantamine of the assay (Table 2).

**Table 2:**
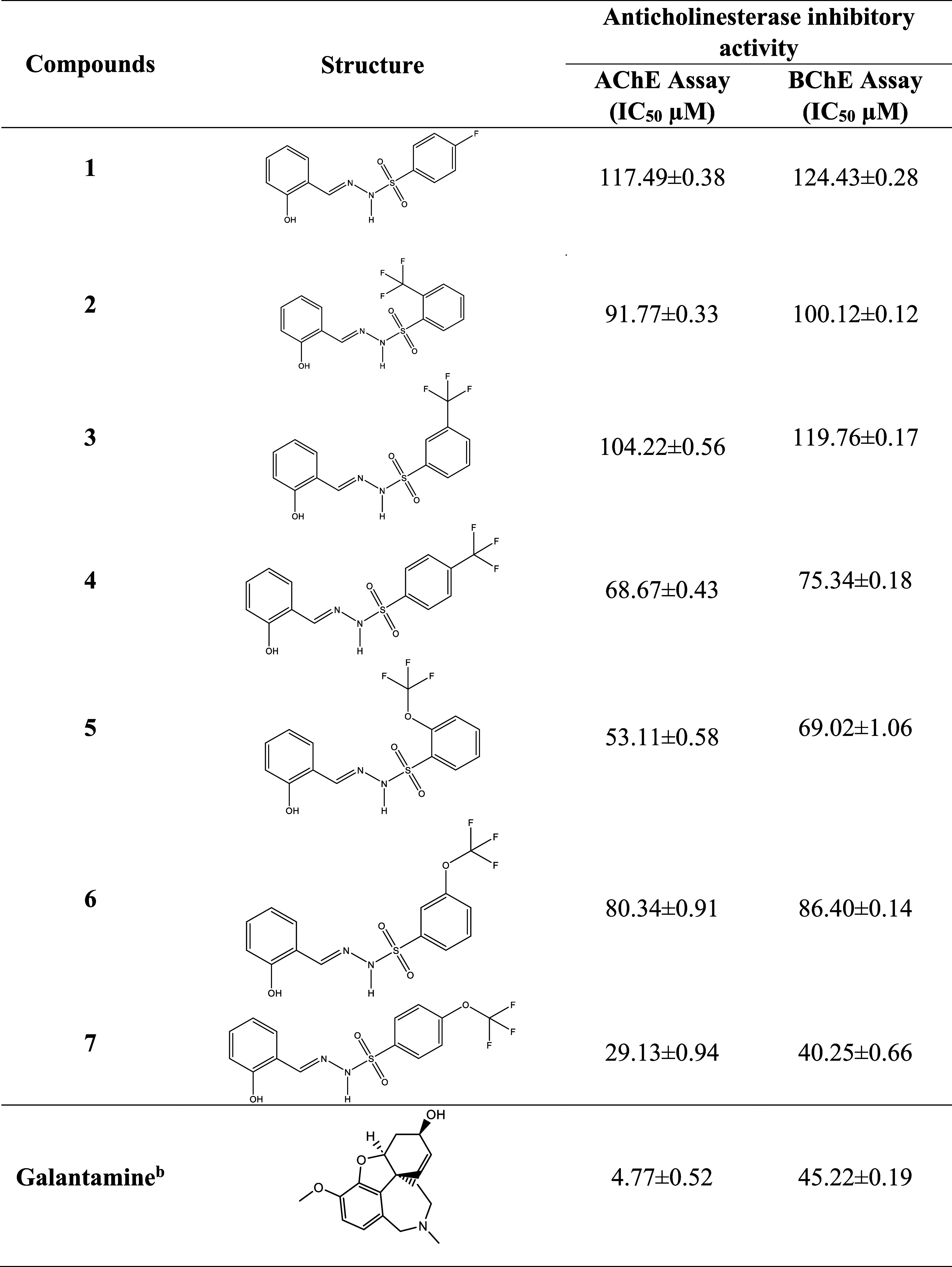
Anticholinesterase Activities of Compounds **1**–**7**

a Values expressed herein are the mean ± SEM of three parallel measurements. *p* < 0.05. NT: not tested.

b Reference compounds.

**Figure 5:**
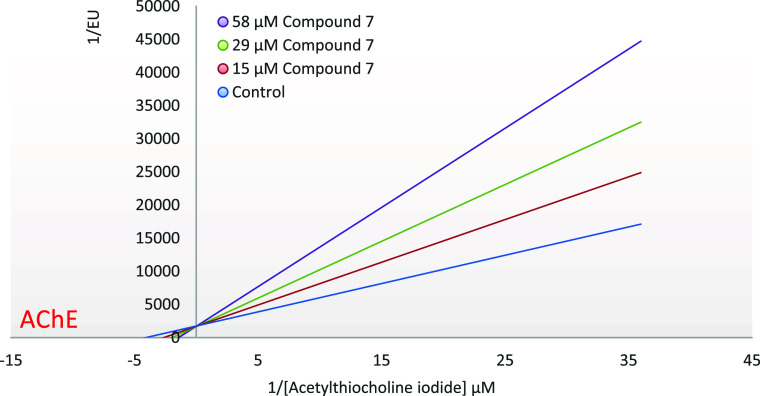
EnzyLineweaver–Burk plot of the inhibition kinetics of AChE by compound **7**.

**Figure 6:**
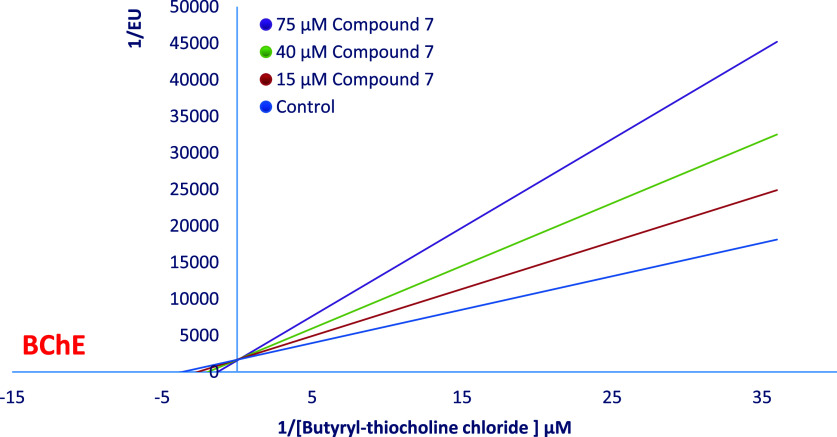
EnzyLineweaver–Burk plot of the inhibition kinetics of BChE by compound **7**.

According to Lineweaver–Burk plots, it was determined that **compound 7** competitively inhibited both AChE and BChE enzymes with values of 28.18 ± 2.51 and 41.74 ± 3.18 μM, respectively (Figure 5, and) (see Figure 6).

#### 4.1.1 Antidiabetic Activities

The antidiabetic inhibition activity of the synthesized hydrazone derivatives (**1**–**7**) was tested against α-amylase and α-glucosidase. Antidiabetic inhibition activity results are given in Table 3. Within the synthesis series, compound **7** (IC_50_: 63.41 ± 0.27 μM) in the α-amylase assay was the most active compound and was determined to be more active than the positive standard acarbose (IC_50_: 79.28 ± 0.55 μM) of the assay. In the α-glucosidase assay, compound **7** (IC_50_: 110.18 ± 0.51 μM) was the most active, followed by **5** (IC_50_: 169.44 ± 0.19 μM), **4** (IC_50_: 171.40 ± 0.75 μM), and **6** (IC_50_: 180.37 ± 1.16 μM), and exhibited higher activity than the positive standard acarbose (IC_50_: 190.14 ± 0.40 μM) of the assay (Table 3).

**Table 3:**
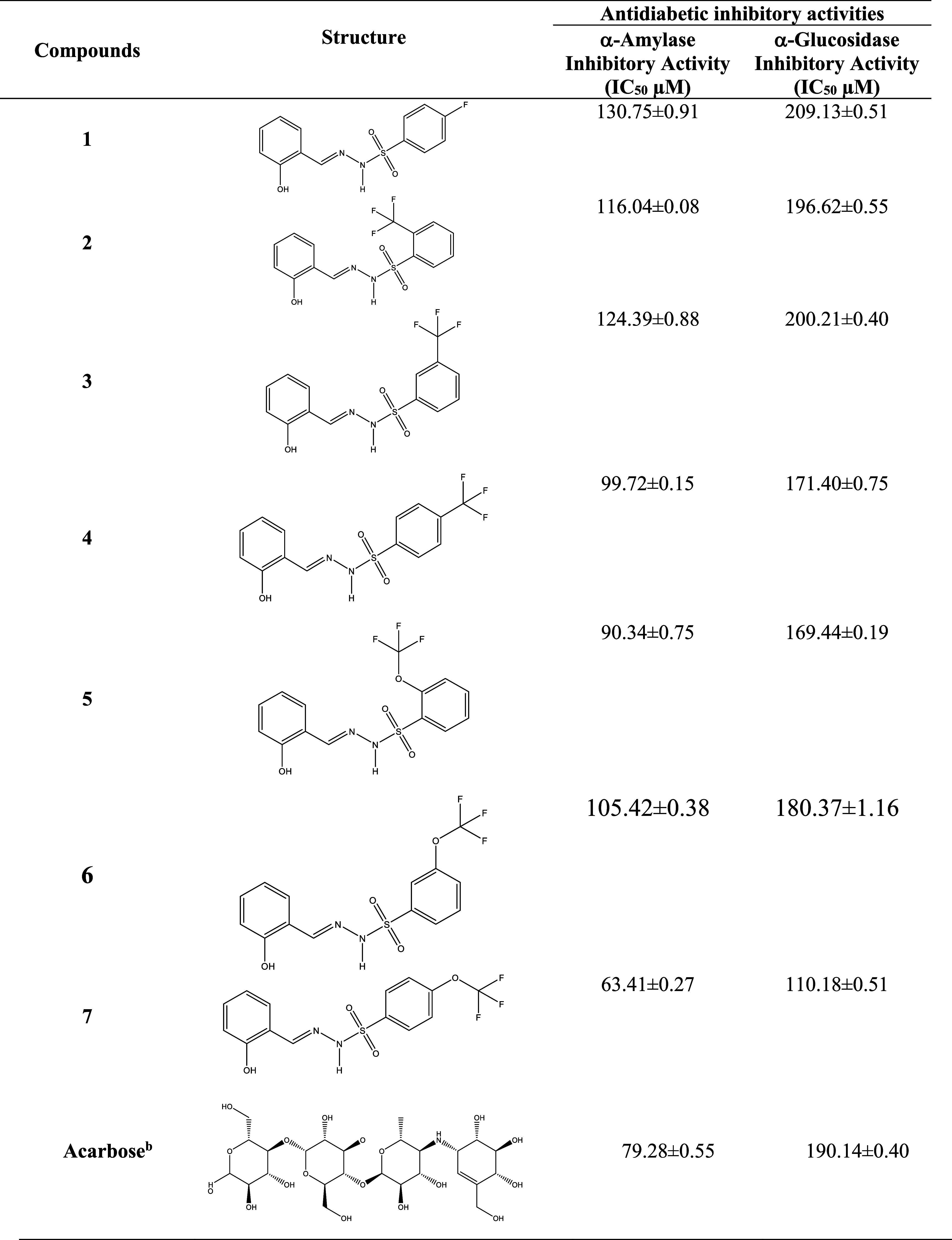
Antidiabetic Activities of Compounds **1–7**

a Values expressed herein are the mean ± SEM of three parallel measurements. *p* < 0.05.

b Reference compounds.

### 4.2 Characterization of the Binding Site of Target Enzymes with Molecular Docking

**Figure 7:**
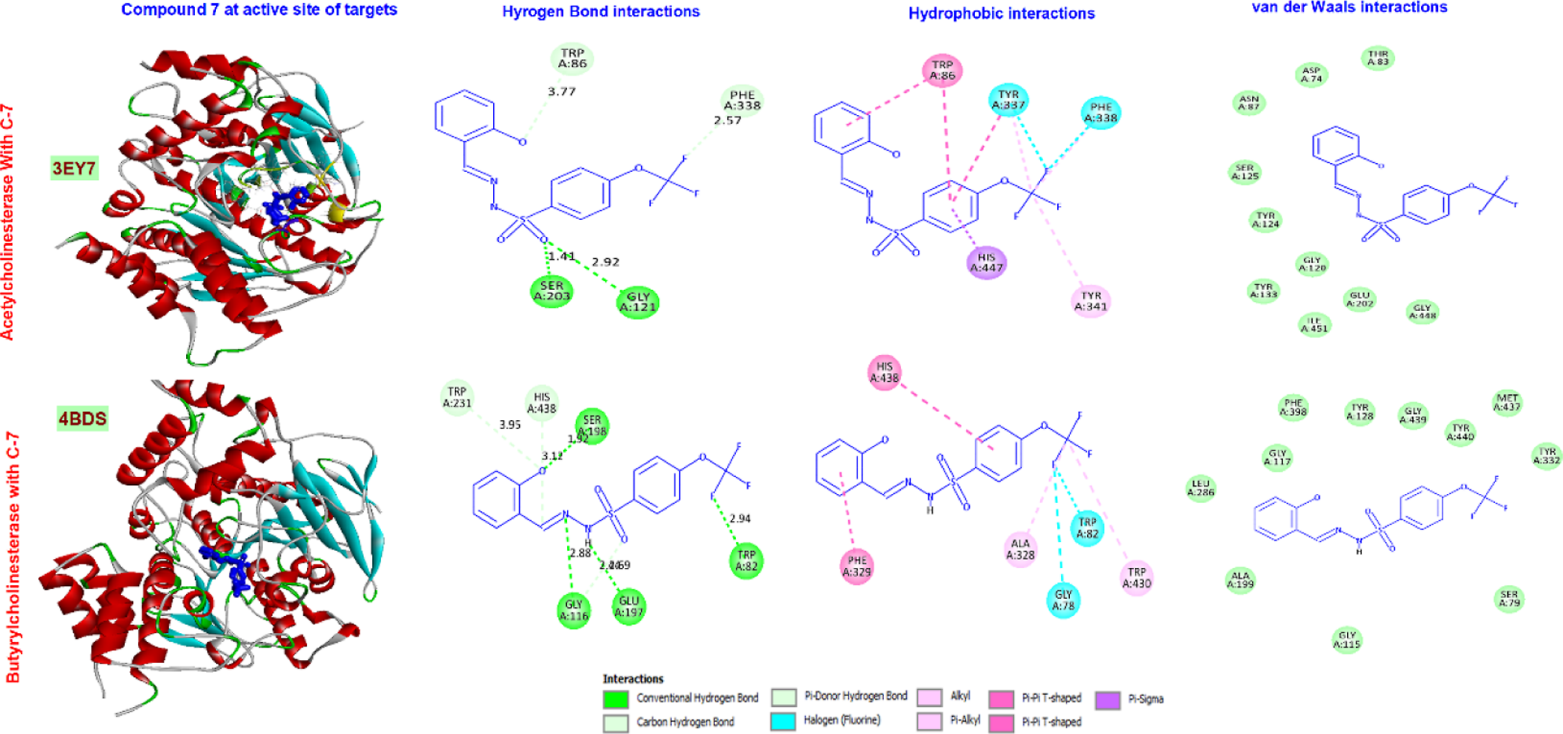
Representation of the interactions and positioning of compounds **7** in the binding site of AChE and BChE.

The results of docking studies performed to show the interaction between enzymes and synthesized molecules are presented in Table 4. In *in vitro* studies, compound **7** was found to have the highest inhibitory potential against all enzymes. Therefore, possible interaction diagrams of that compound with the enzymes were prepared. The binding modes of compound **7** to AChE and BChE are given in Figure 7. The molecule bonded to the AChE enzyme with a MolDock score of −142.519 and interacted with amino acids Ser203 and Gly121 through normal hydrogen bonds and with residues Trp86 and Phe338 through carbon hydrogen bond interactions. While Tyr337 and Phe338 make halogen bond interactions with the fluorines on the molecule, there are Pi–Pi T-shaped interactions between the aromatic rings on the molecule and Trp86 and Tyr337 residues. Compound **7** made alkyl interactions with Tyr341 and Tyr337 and a Pi–Sigma interaction with His447. Compound **7** made hydrogen bonds in very close proximity with Ser203, one of the important amino acids of the catalytic triad, and made a Pi-sigma interaction with His447. It also communicates with Tyr337, located in the phosphyl site, through multiple types of interactions.^40^ It can be said that these interactions contribute significantly to the inhibitory potential of the molecule on the acetylcholine enzyme activity.

**Table 4:** Docking Results of Synthesized Molecules against AChE, BChE, α-Amylase, and α-Glucosidase

	AChE	BChE	α-amylase	α-glucosidase
compound	MolDock score	MolDock score	MolDock score	MolDock score
1	–133.097	–113.206	–106.566	–113.471
2	–140.202	–124.457	–111.526	–116.67
3	–144.916	–129.687	–112.558	–120.386
4	–139.612	–127.474	–115.968	–115.297
5	–143.421	–129.269	–109.432	–122.497
6	–156.094	–130.121	–113.096	–121.398
7	–142.519	–124.908	–117.344	–113.275

The affinity of compound **7** toward the BChE was found to have a −124.908 MolDock score. The molecule interacted with Gly116, Glu197, Trp82, and Ser198 residues via hydrogen bonding. While it makes carbon–hydrogen bonds with amino acids Trp231 and His438, it makes halogen bonds with the fluorine atom. While fluorines had alkyl interactions with amino acids Ala328 and Trp430, aromatic rings contributed to Pi–Pi T-shaped interactions. It is observed that BChE makes both close hydrogen bonds and hydrophobic interactions with two of the residues Ser198, His438, and Glu325, which form the catalytic triad.^41^ It can be said that these bonds are important in the inhibitory effect of the molecule on the enzyme.

**Figure 8:**
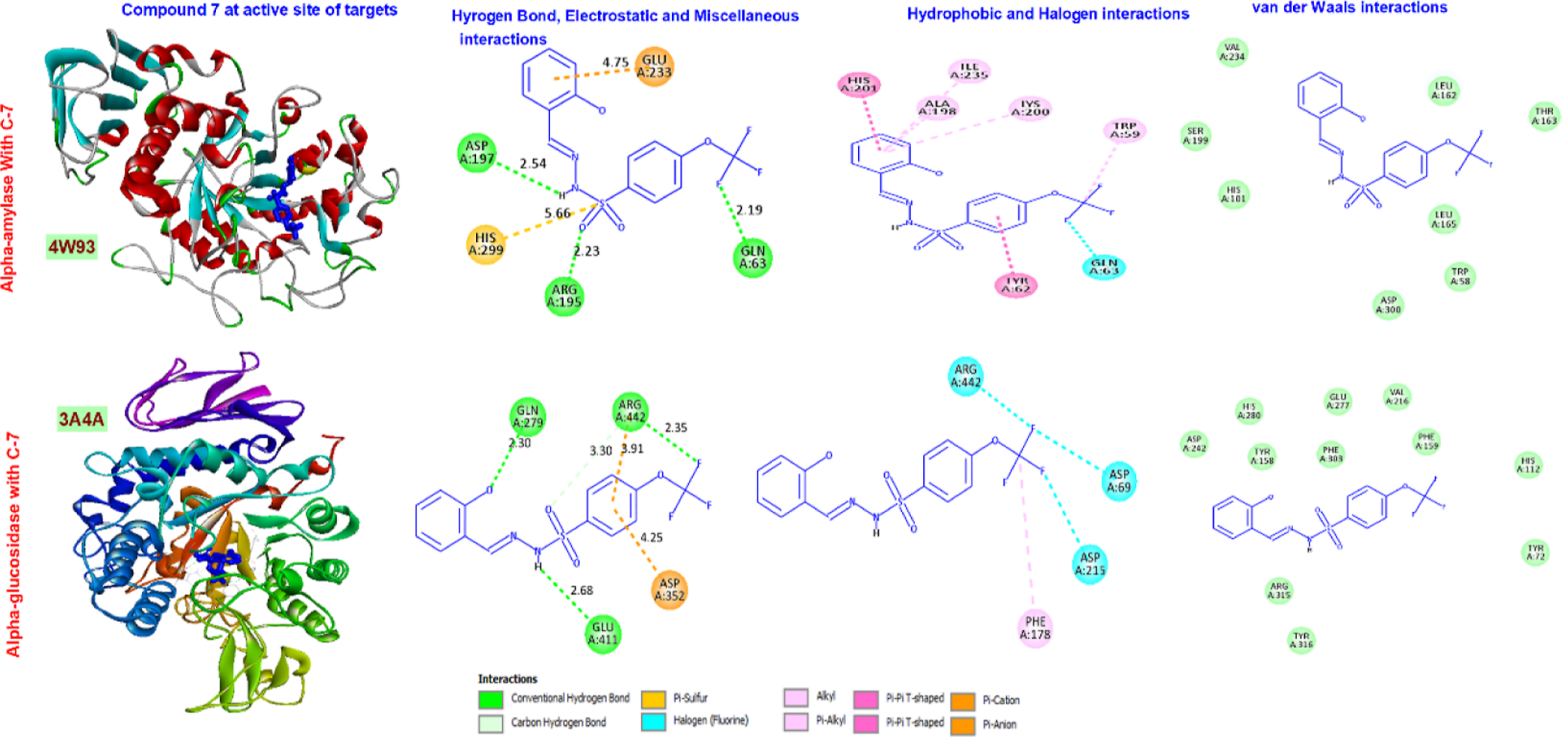
Representation of the interactions and positioning of compounds **7** in the binding site of α-glucosidase and α-amylase.

The docking scores of compound **7** against α-amylase and α-glucosidase were found to be −117.344 and −113.275 MolDock, respectively. Details of the interaction with amino acids in the active site of enzymes are presented in Figure 8.

Compound **7** formed three hydrogen bonds with Asp197, Arg195, and Gln63 residues found in α-amylase. It showed the highest number of hydrophobic interactions (alkyl: Ile235, Ala298, Lys200, Trp59; Pi–Pi T-shaped) compared to other compounds besides electrostatic interactions (halogen: Gln63, Pi-cation: Glu233). It demonstrated Pi-sulfur interactions via His299. It can be said that the molecule has a low inhibition effect due to its weak interaction with important amino acids such as Asp197, Glu233, and Asp300.^40^

Compound **7** constructed hydrogen bonds with Gln297, Arg442, and Glu411. It conducted alkyl interactions with Phe178. It showed three halogen interactions with Adg442, Asp69, and Asp215. It established a Pi-cation interaction with Asp352. The molecule interacted only with Asp352, one of the catalytic residues, and did not interact with other amino acids, such as Asp215 and Glu277. The reason for the low inhibition effect may be that it does not interact strongly enough with important amino acids involved in the catalytic process.^41^

#### 4.2.1 In Silico Studies

*In silico* studies provide important information about the design of the molecule and its potential as a drug candidate. *In silico* studies enabled the calculation of absorption, distribution, metabolism, and excretion (ADME properties, basic parameters affecting drug metabolism, and computational toxicity risk parameters (mutagenic effect, tumorigenic effect, irritating effect, and reproductive effect). When ADME values were examined, the molecular weights of fluorinated sulfonyl hydrazone compounds were found to be in the range of 294.30–360.31 g/mol (150 g/mol < MA < 500 g/mol). Topological polar surface area (TPSA A^2^) values were found to be in the range of 41.4687.12–96.37 A^2^ in compounds **1–7** (TPSA < 70 A^2^). Compounds **1–7** were found to have iLog P values of 1.23–2.2.20. It was determined that any of the compounds do not cross the blood–brain barrier; therefore, they will not harm the central nervous system; it is expected not to cause depression and drowsiness (Table 5). It was determined that none of the compounds have mutagenic, tumorigenic, irritating, or reproductive effects. This is a positive feature for a drug candidate (Table 6).^42^

**Table 5:**
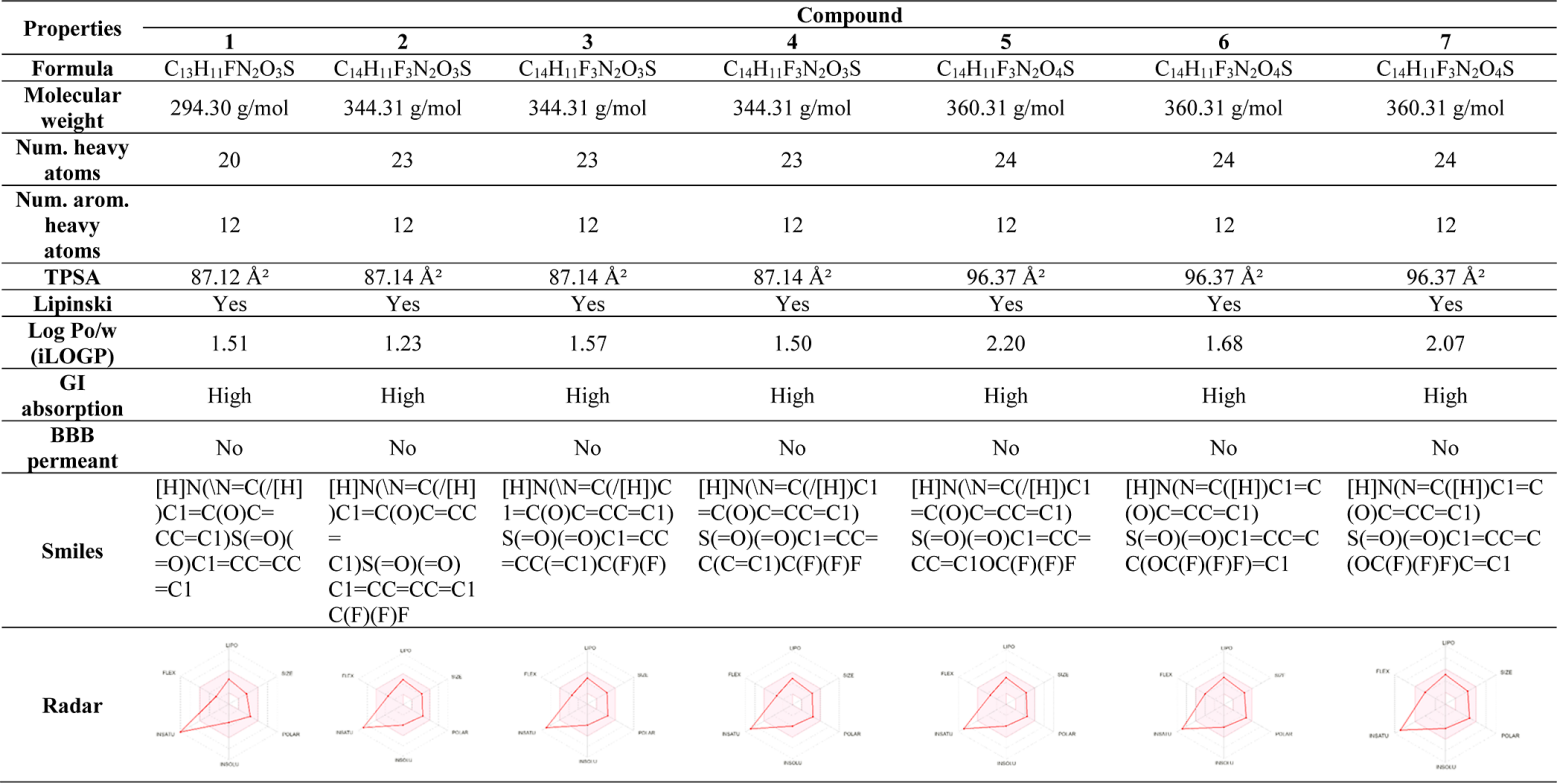
Drug-Likeness Properties and Bioavailability Radar of Fluorinated Sulfonyl Hydrazone Compounds with the Methyl Group (**1–7**)

**Table 6:** Toxicity Risks of Fluorinated Sulfonyl Hydrazones (**1**–**7**)

compound	toxicity risks			
	mutagenic	tumorigenic	irritant	reproductive effective
1	no risk	no risk	no risk	no risk
2	no risk	no risk	no risk	no risk
3	no risk	no risk	no risk	no risk
4	no risk	no risk	no risk	no risk
5	no risk	no risk	no risk	no risk
6	no risk	no risk	no risk	no risk
7	no risk	no risk	no risk	no risk

## 5 Conclusions

All compounds were synthesized in pure form in the range of 20–56% yield. After structure characterization, the anticholinesterase inhibition activity (AChE and BChE) and antidiabetic inhibition activities (α-amylase and α-glucosidase) were examined. When anticholinesterase inhibition (AChE and BChE) activities were examined, it was observed that compound **7** had higher BChE inhibition activity than galantamine, which is used as a standard, and AChE showed seven times lower inhibitory activity than the standard. It was determined that compound **7** showed selectivity toward BChE inhibition activity. When antidiabetic activities (α-amylase and α-glucosidase) were examined, in α-amylase inhibitory activity, all 7 compounds exhibited lower activity than galantamine, which is used as a standard. In α-glucosidase inhibitor activity, the observed levels of activities were as **7** > **5** > **4** > **6**>galantamine>**2** > **3**>**1**. Compound **7** was the most active compound in both α-amylase and α-glucosidase activities. When the water solubility of all compounds was examined, it was determined that all compounds were moderately soluble (Table S1).^32^

## Supporting Information Available

^1^H NMR, ^13^C NMR, and FTIR spectra of compounds **1**–**7**; HSQC NMR, COSY NMR, HMBC NMR, and ^19^F NMR spectra of compound **6**; and water solubility of compounds **1**–**7** (PDF)
