# Supplementary material for: Design, Synthesis, Anticholinesterase and Antidiabetic Inhibitory Activities, and Molecular Docking of Novel Fluorinated Sulfonyl Hydrazones
Source: ACS Omega. 2024 Sep 25;9(40):42037–48. doi: 10.1021/acsomega.4c07160 (PMC11465619; doi:10.1021/acsomega.4c07160)
Supplement: Supplementary file 1 — ao4c07160_si_001.pdf [file ao4c07160_si_001.pdf]

**SUPPORTING INFORMATION**

# Design, Synthesis, Anticholinesterase and Antidiabetic Inhibitory Activities and Molecular Docking of Novel Fluorinated Sulfonyl Hydrazones

Bedriye Seda Kurşun Aktar[a]

[a]Department of Hair Care and Beauty Services, Yeşilyurt Vocational School, Malatya Turgut Özal University, Malatya, Turkey

Email: bseda.kursunaktar@ozal.edu.tr

Corresponding Author: Bedriye Seda Kurşun Aktar

Address: Malatya Turgut Özal University

Yesilyurt Vocational School

Ikizce District Ikizce Kümeevler No 100/4

44900 Yesilyurt Malatya

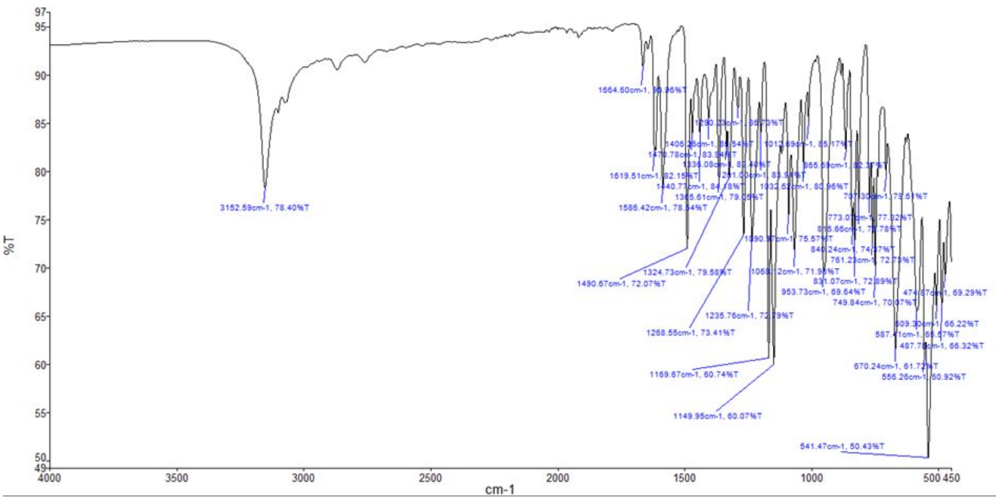

**Fig. S1**. FTIR spectrum of compound **1**

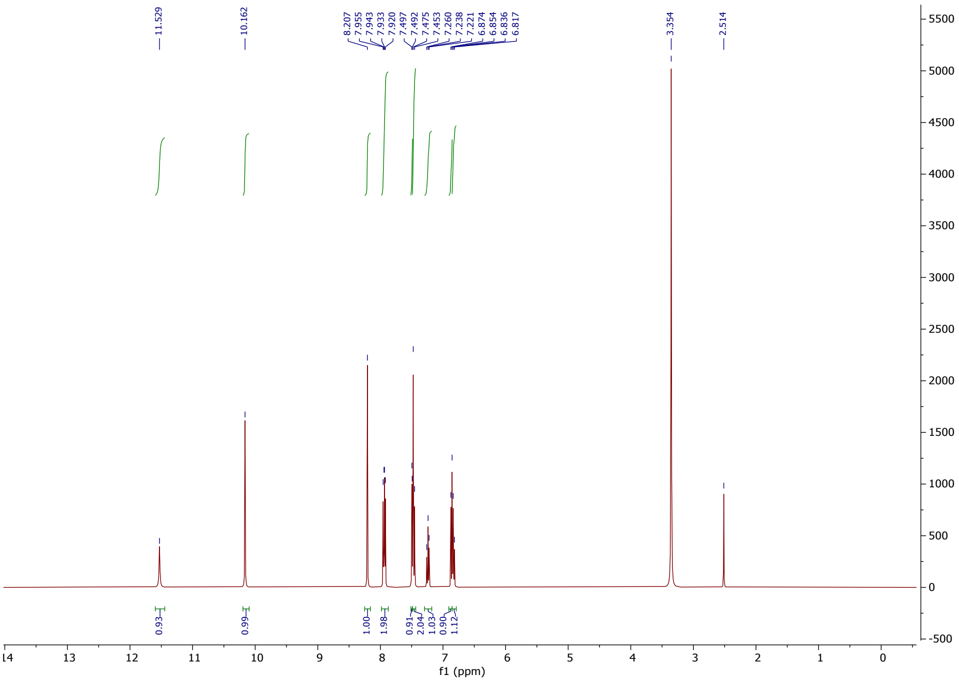

**Fig. S2.** $^1$H NMR spectrum of compound **1**

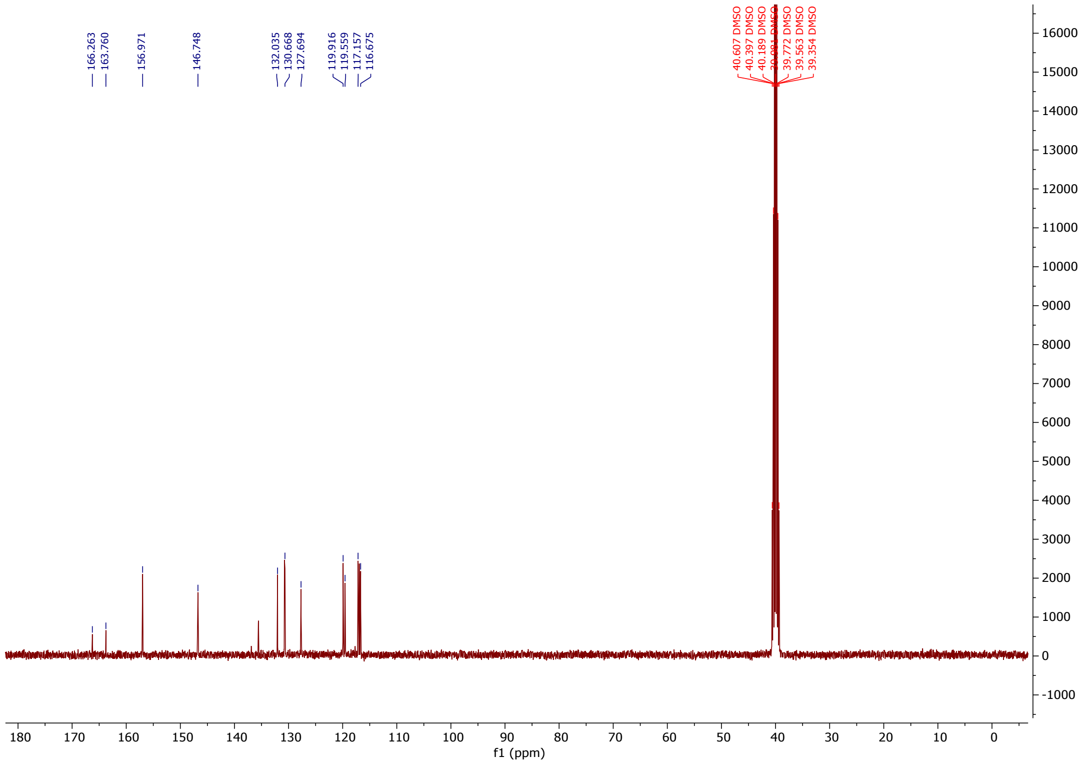


**Fig. S3.** $^{13}C$ NMR spectrum of compound **1**

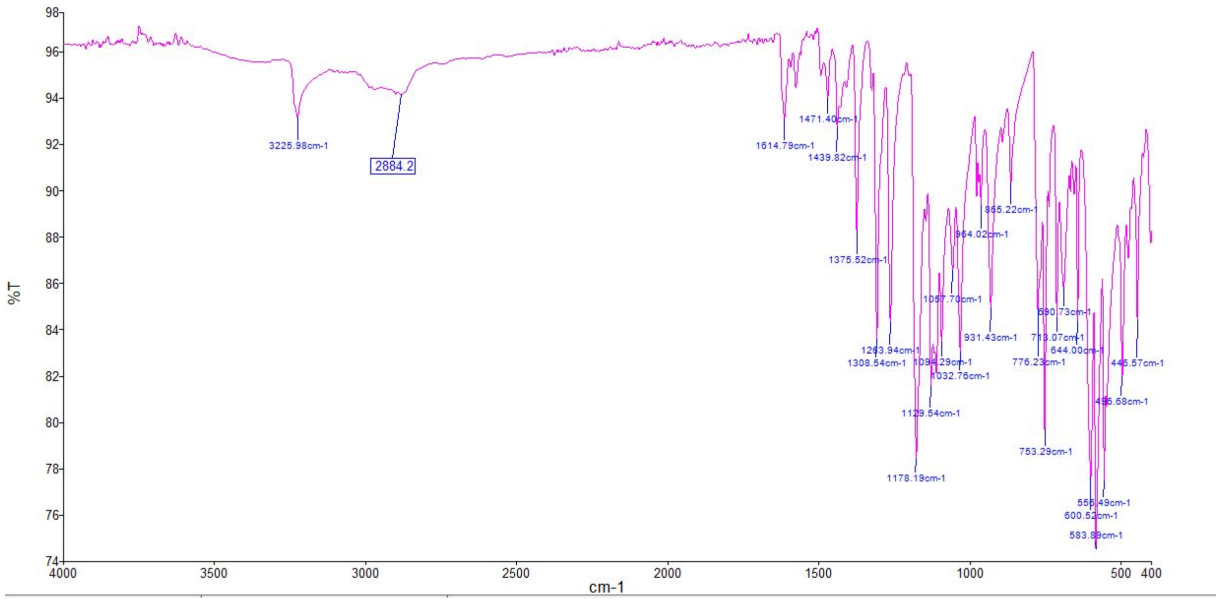


**Fig. S4**. FTIR spectrum of compound **2**

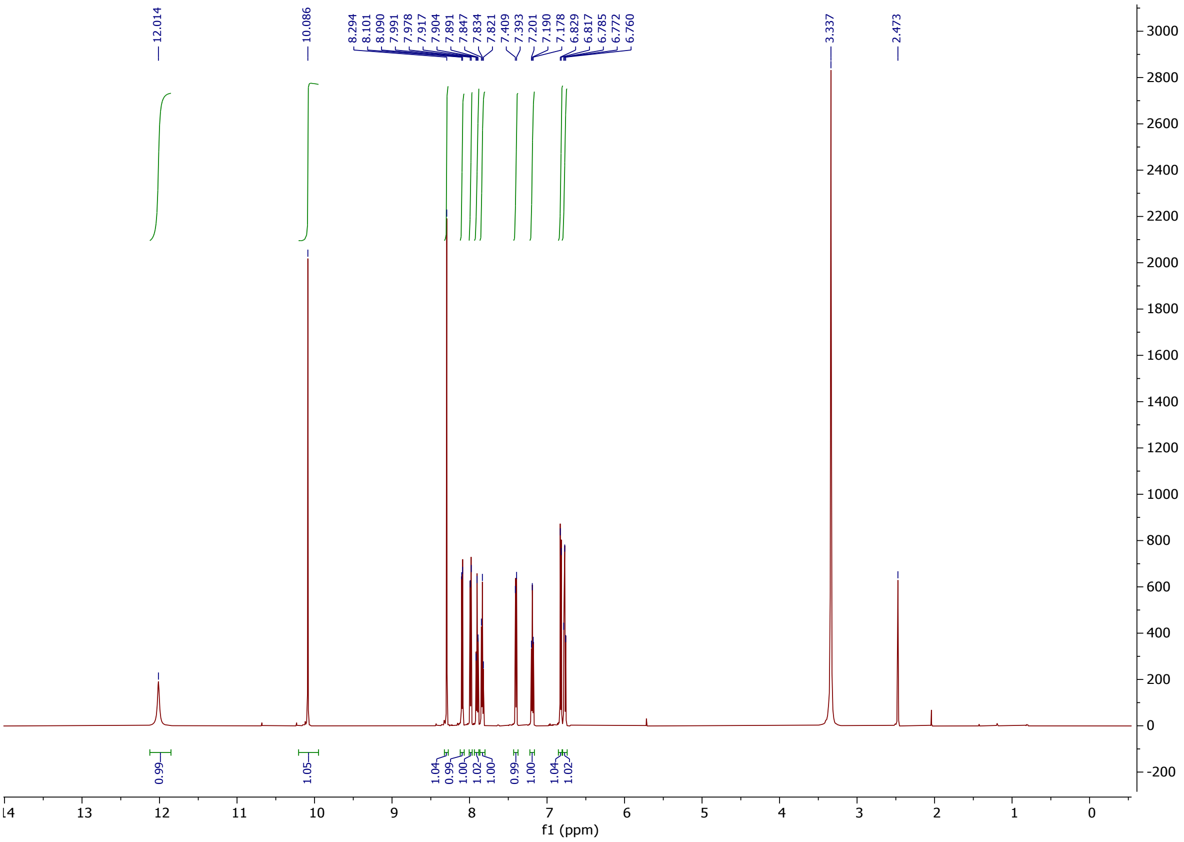


**Fig. S5.** $^{1}$H NMR spectrum of compound **2**

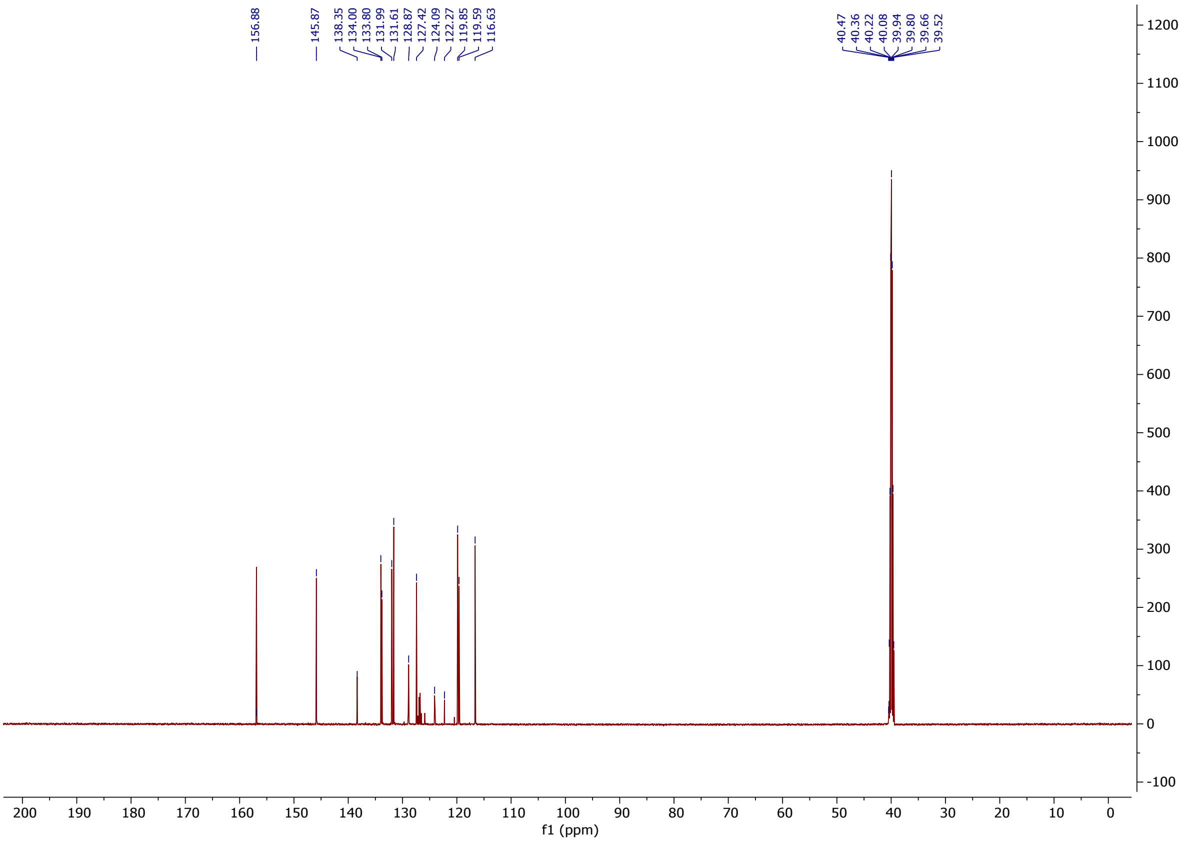


**Fig. S6.** $^{13}$C NMR spectrum of compound **2**

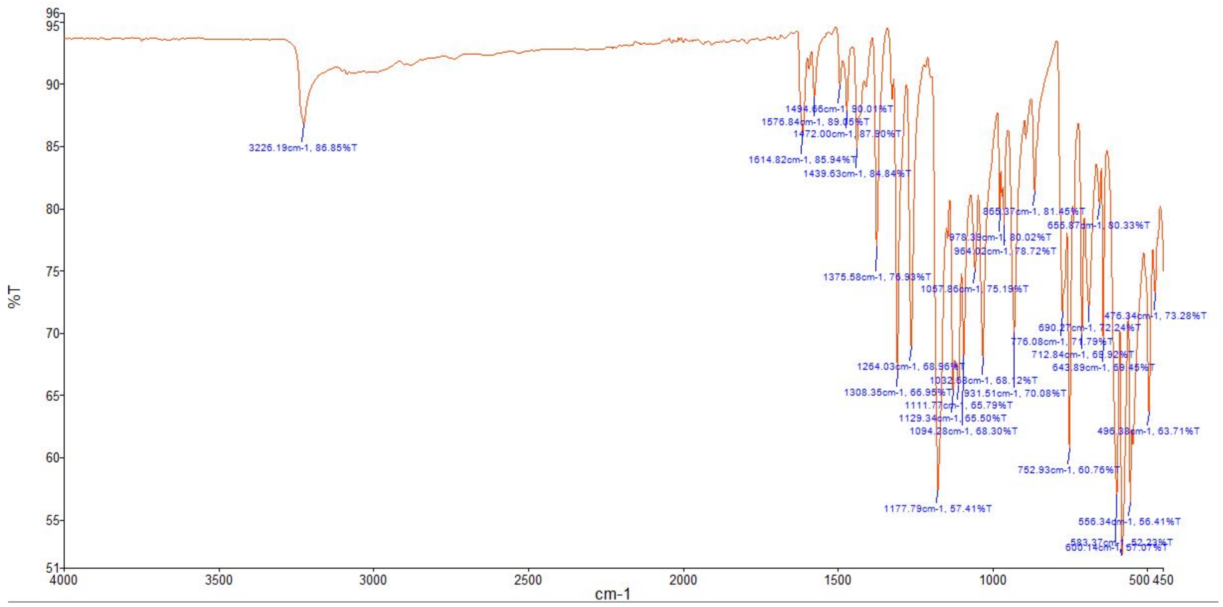

**Fig. S7**. FTIR spectrum of compound **3**

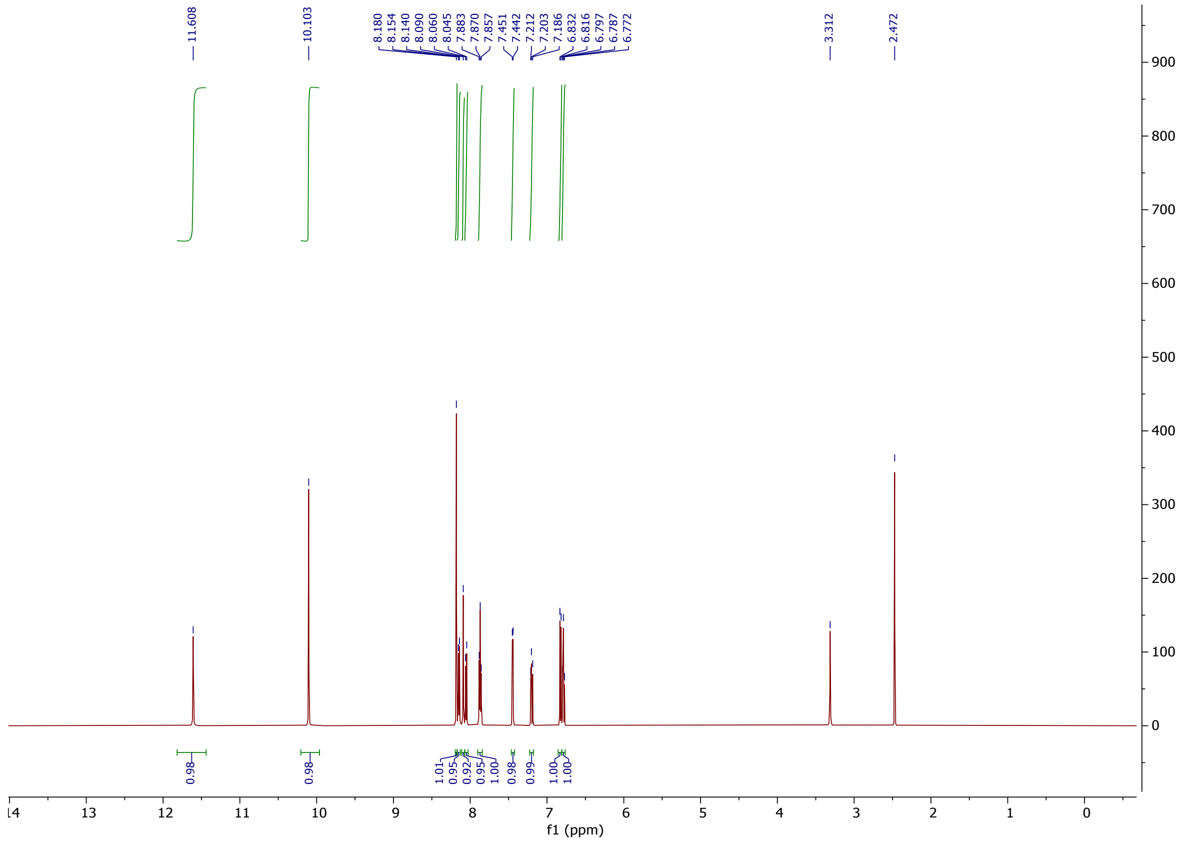

**Fig. S8.** $^{1}$H NMR spectrum of compound **3**

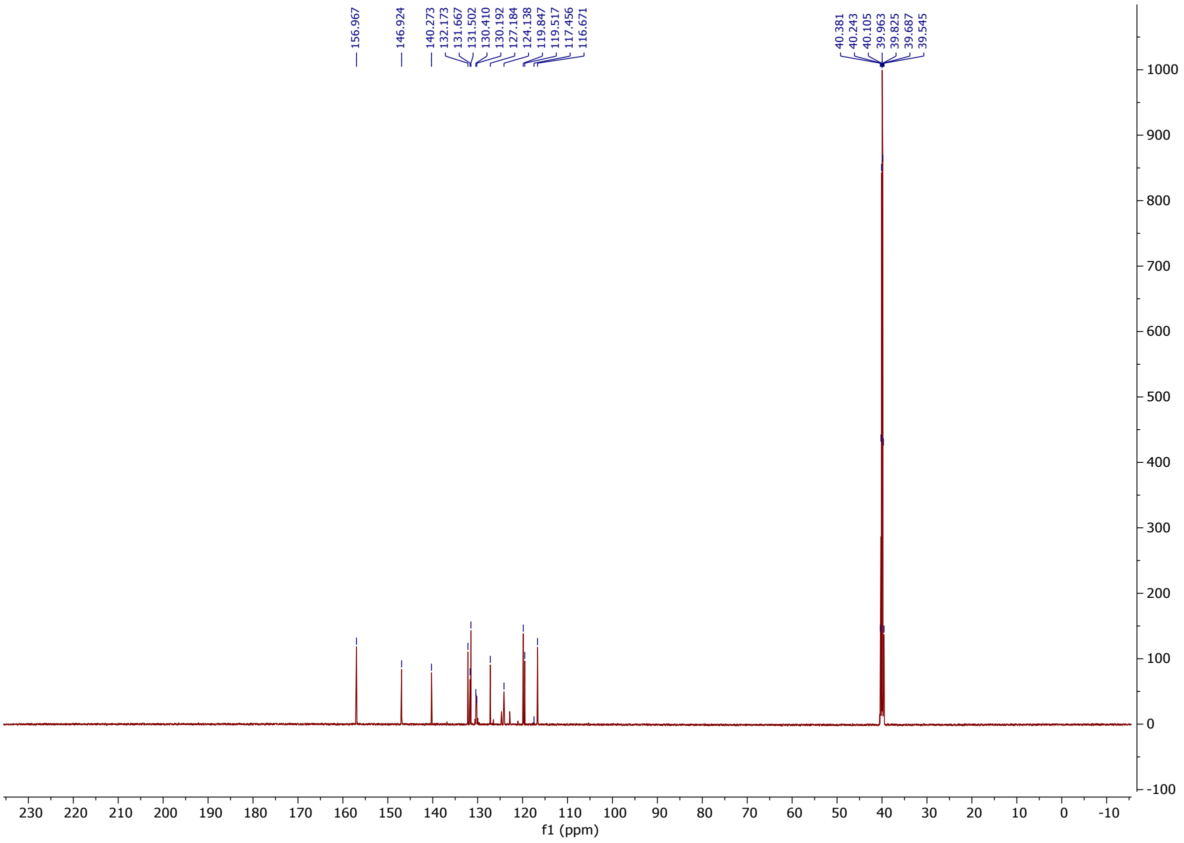


**Fig. S9.** $^{13}$C NMR spectrum of compound **3**

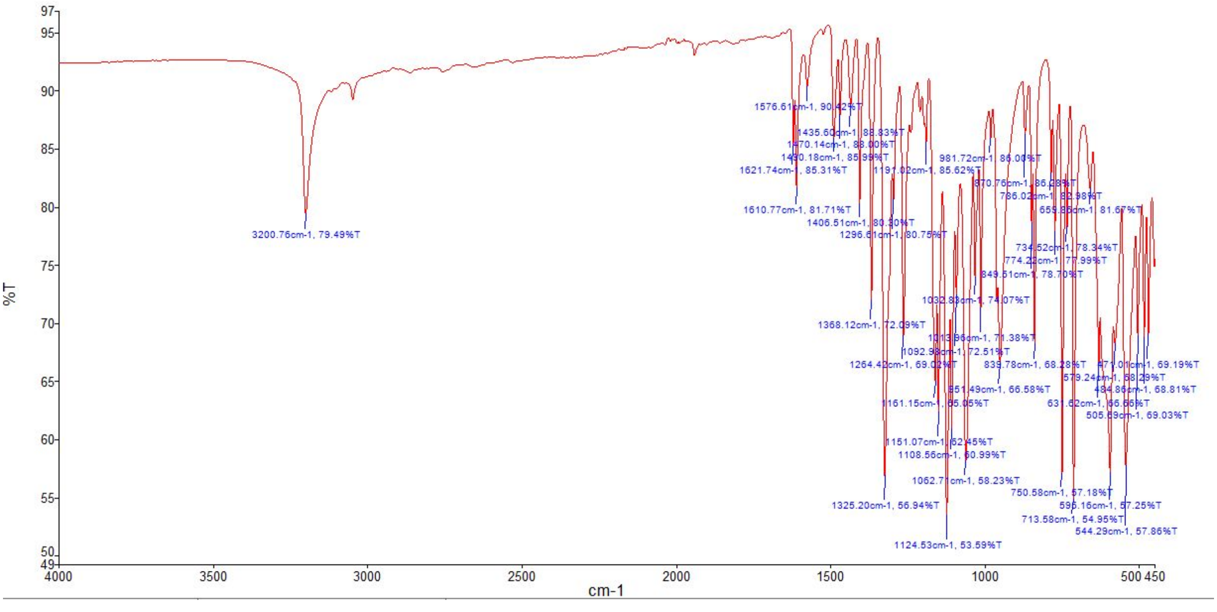


**Fig. S10**. FTIR spectrum of compound **4**

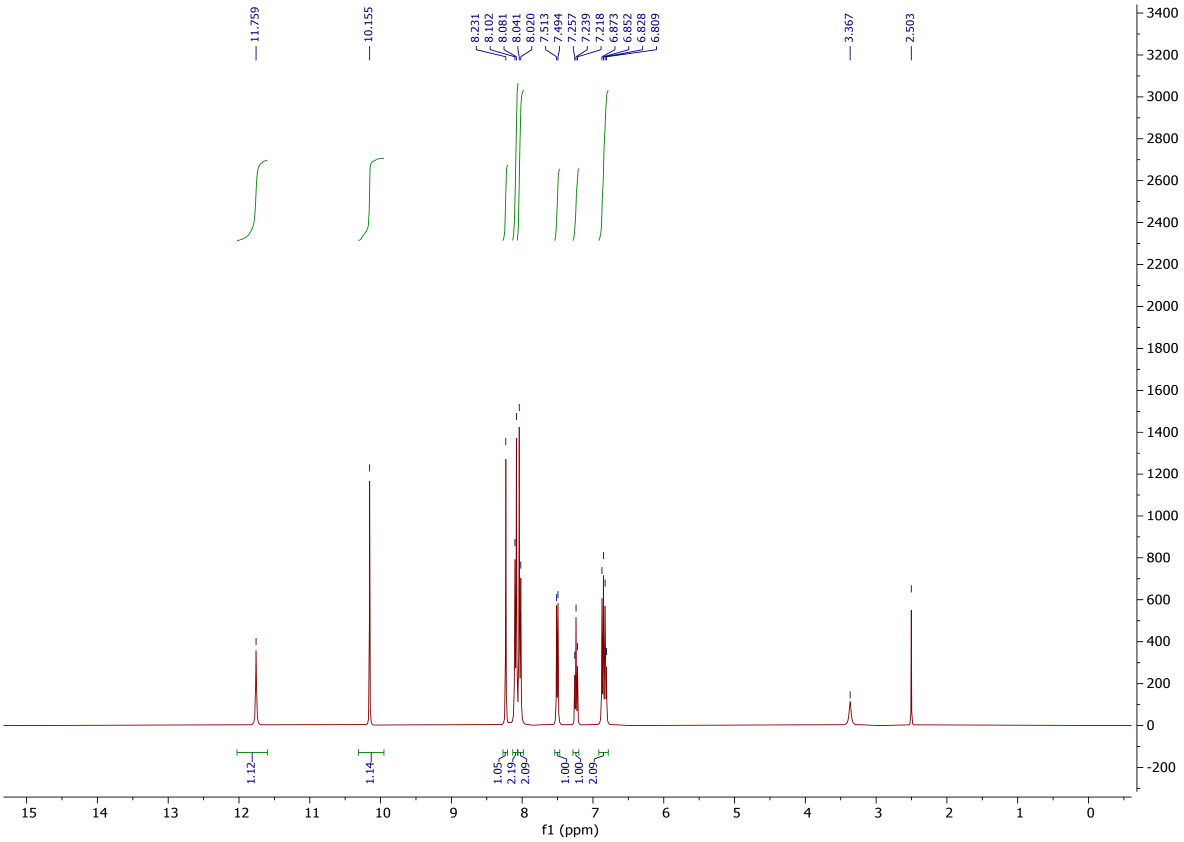

**Fig. S11.** $^{1}H$ NMR spectrum of compound **4**

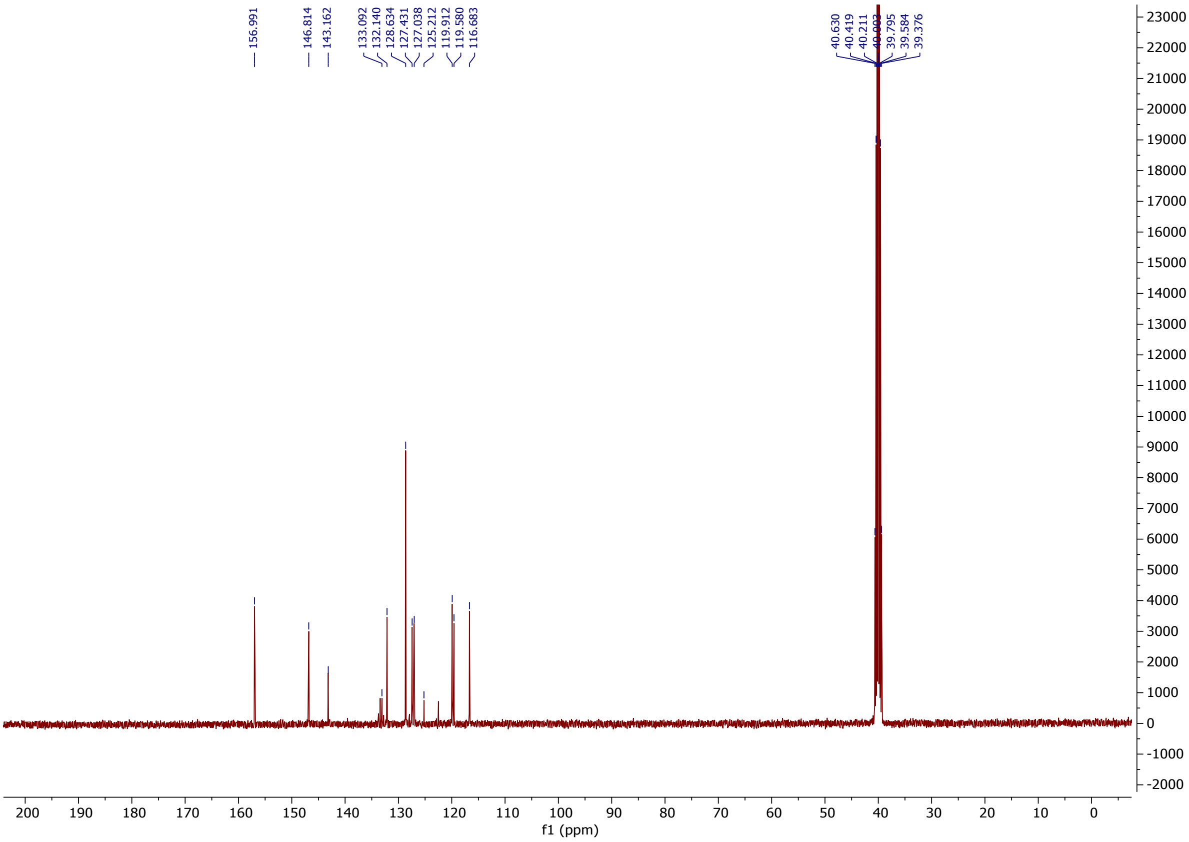

**Fig. S12.** $^{13}C$ NMR spectrum of compound **4**

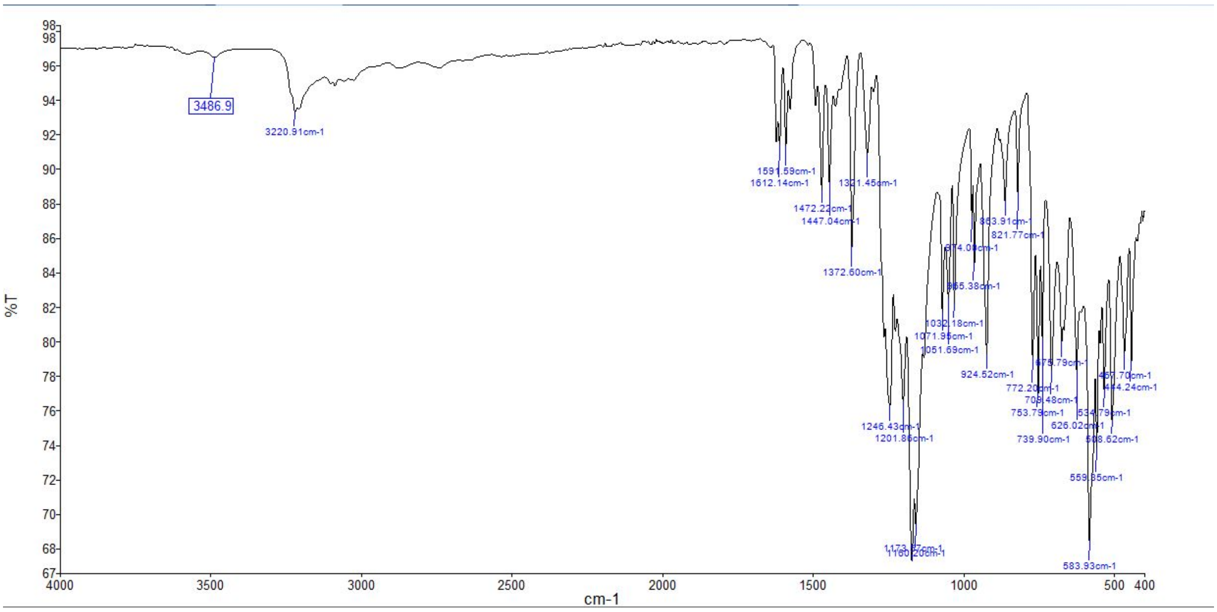

**Fig. S13**. FTIR spectrum of compound **5**

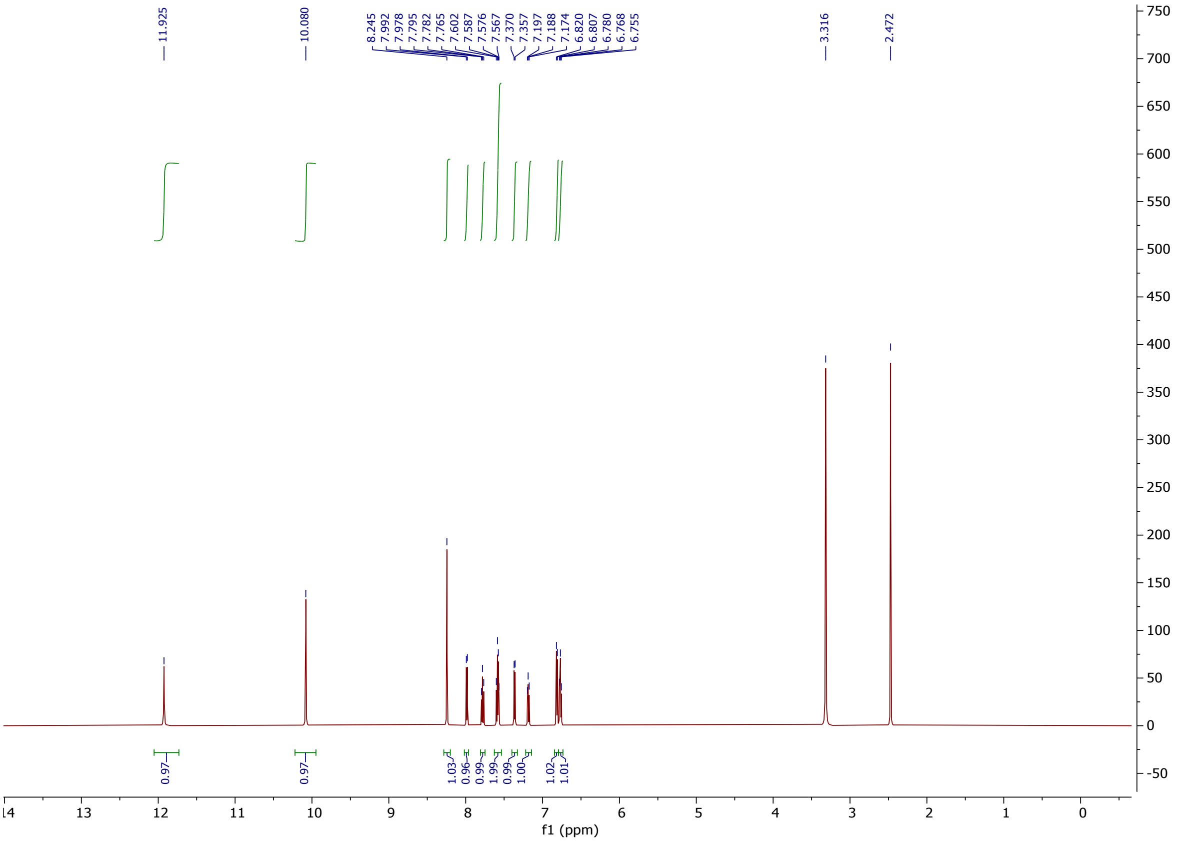

**Fig. S14.** $^{1}$H NMR spectrum of compound **5**

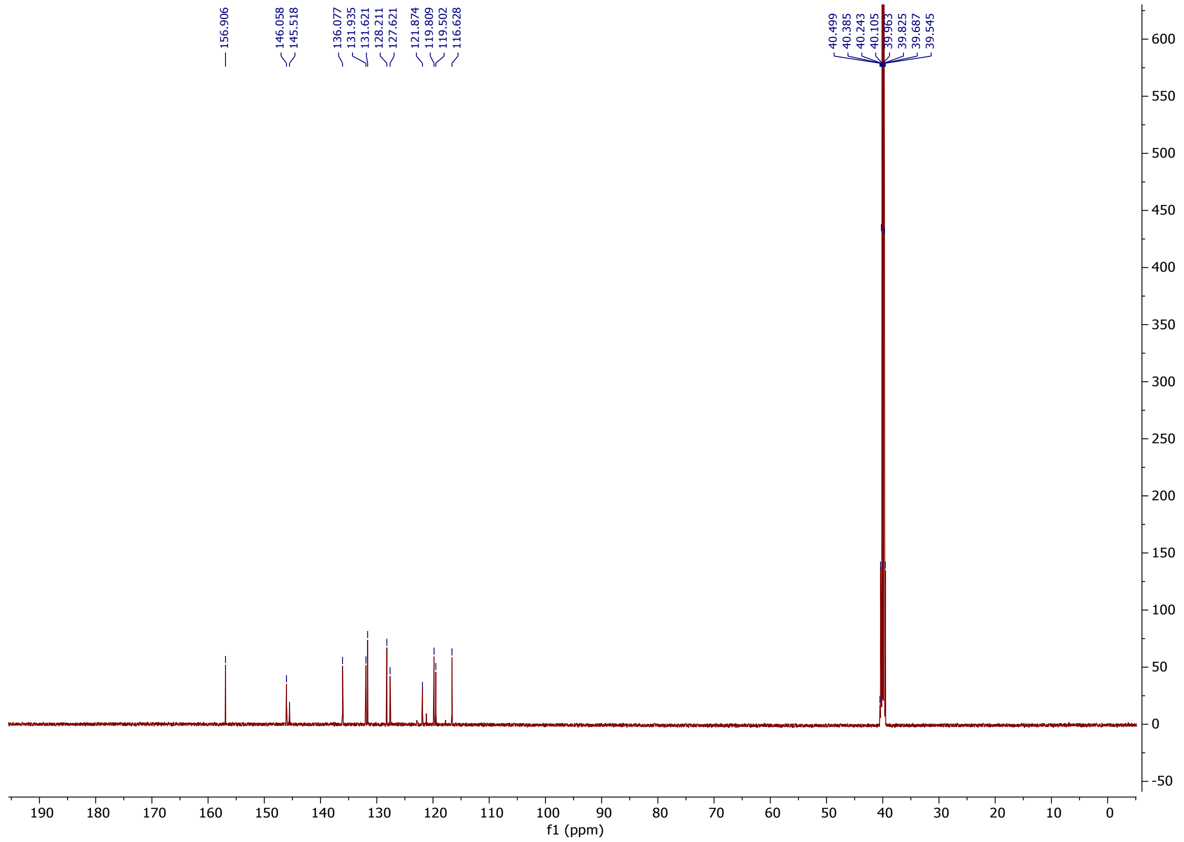


**Fig. S15.** $^{13}$C NMR spectrum of compound **5**

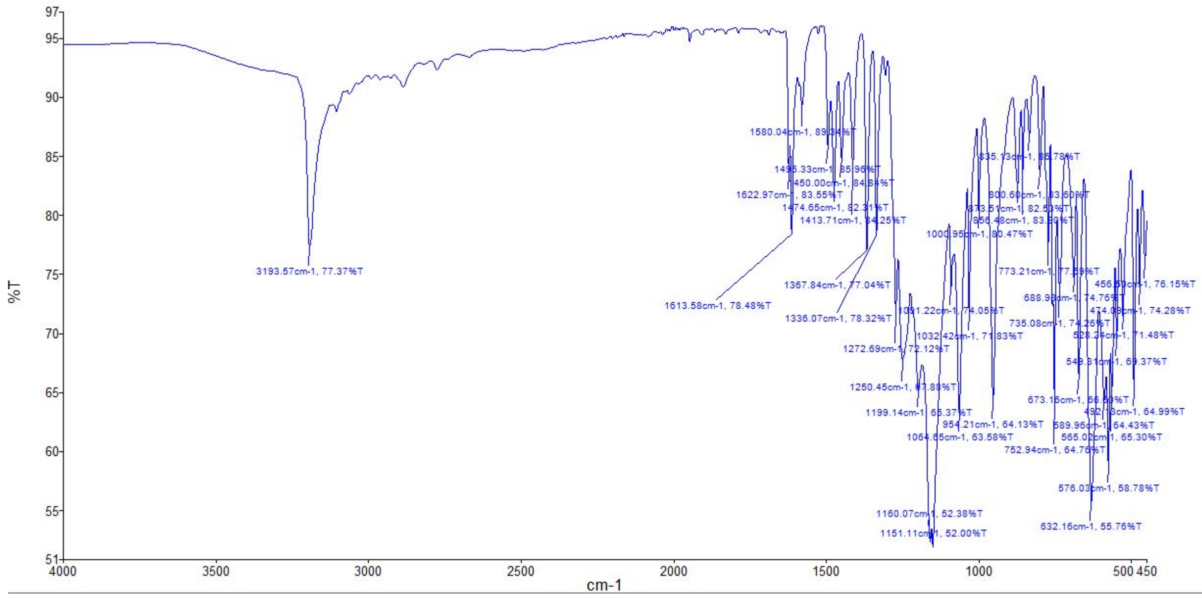

**Fig. S16**. FTIR spectrum of compound **6**

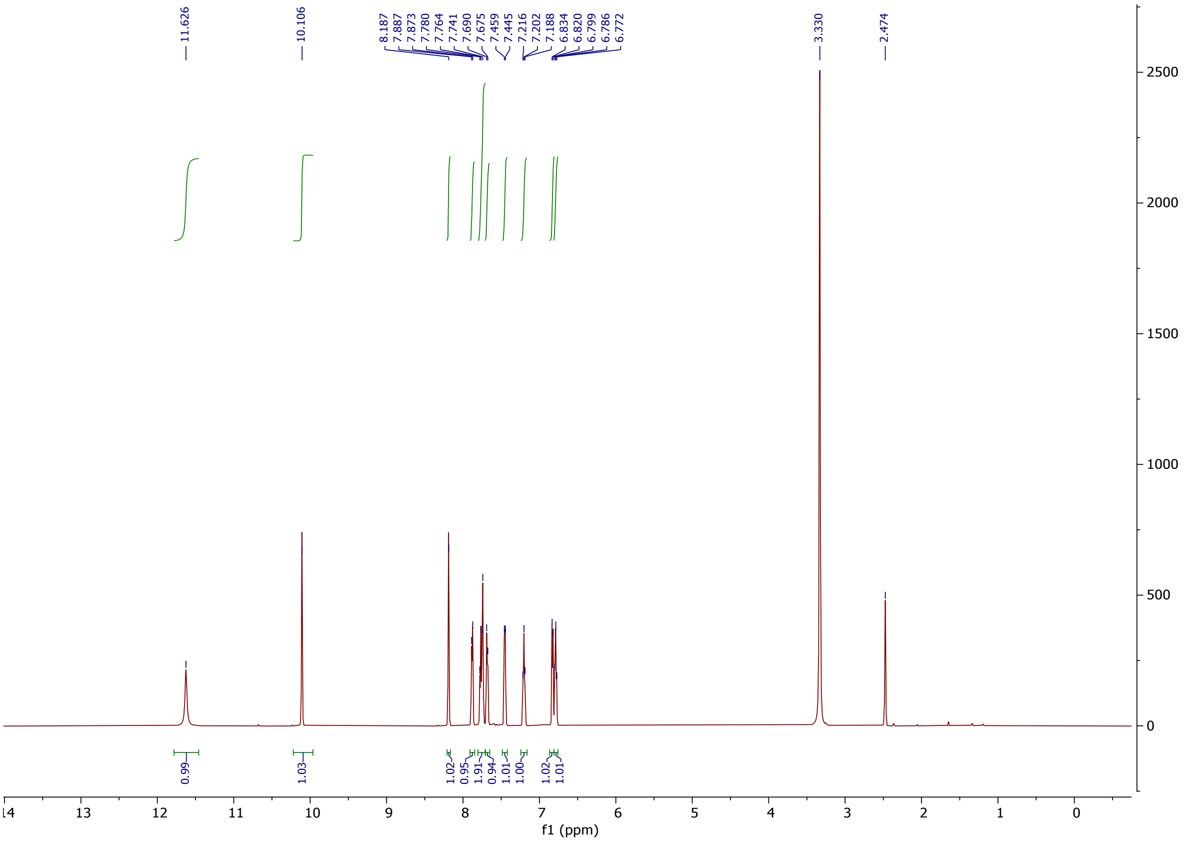


**Fig. S17.** $^{1}H$ NMR spectrum of compound **6**

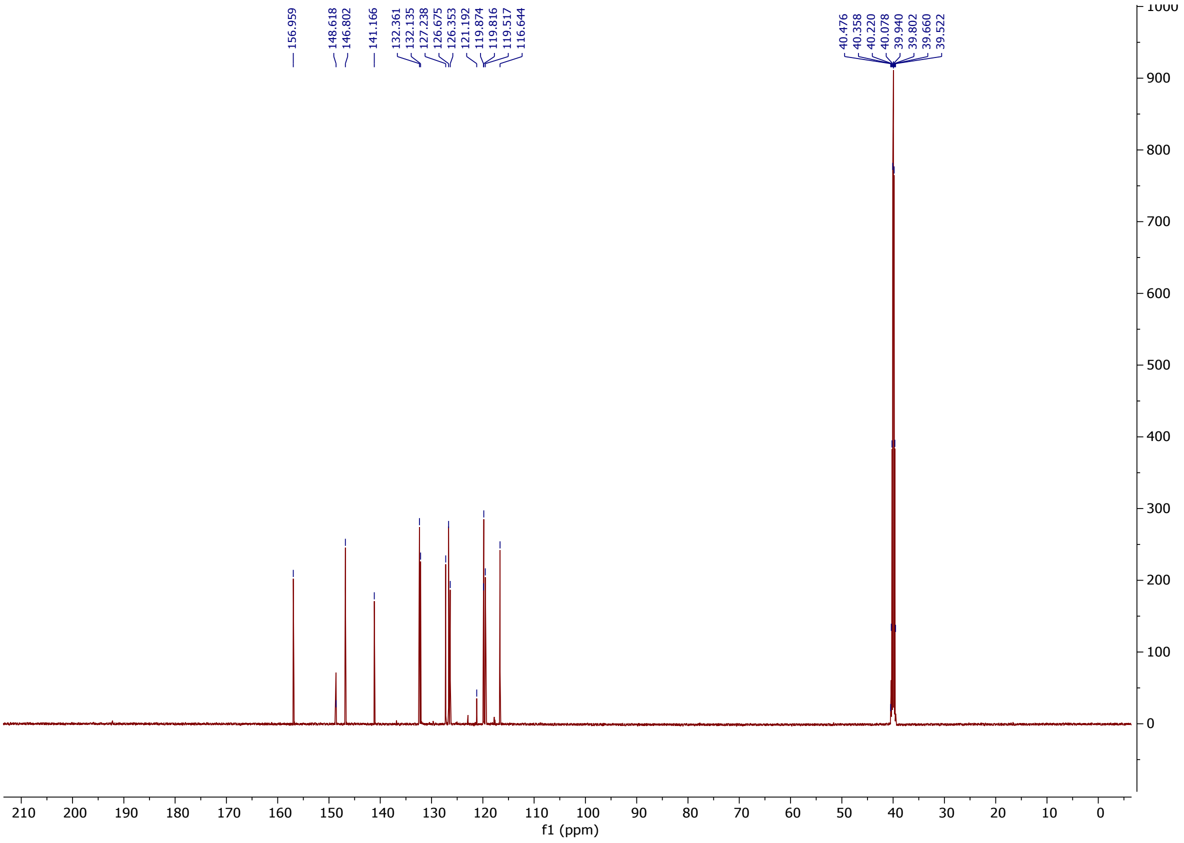


**Fig. S18.** $^{13}C$ NMR spectrum of compound **6**

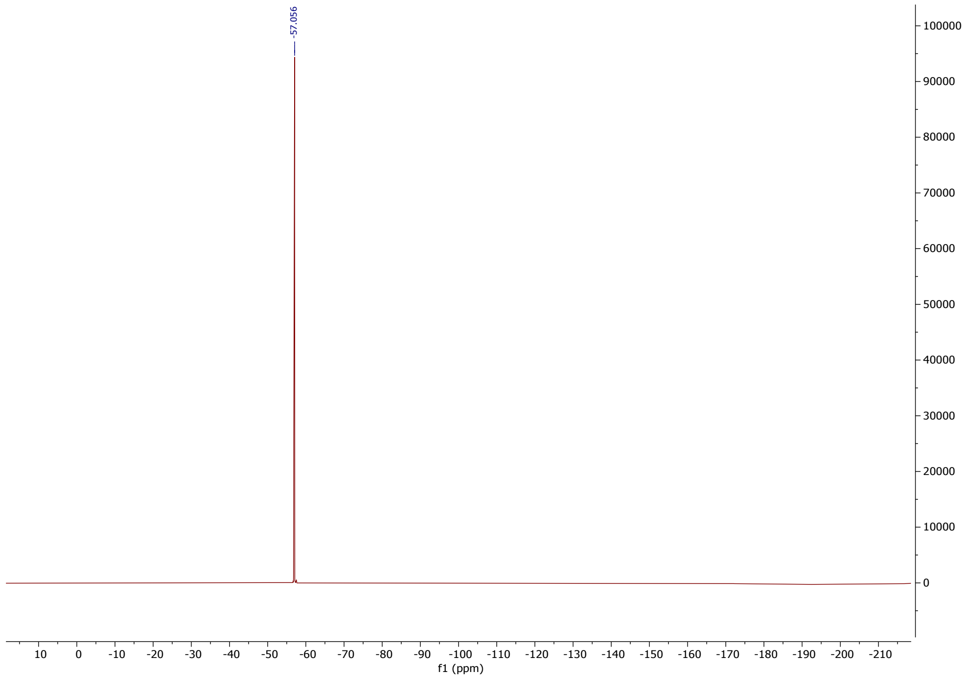


**Fig. S19.** $^{19}$F NMR spectrum of compound **6**

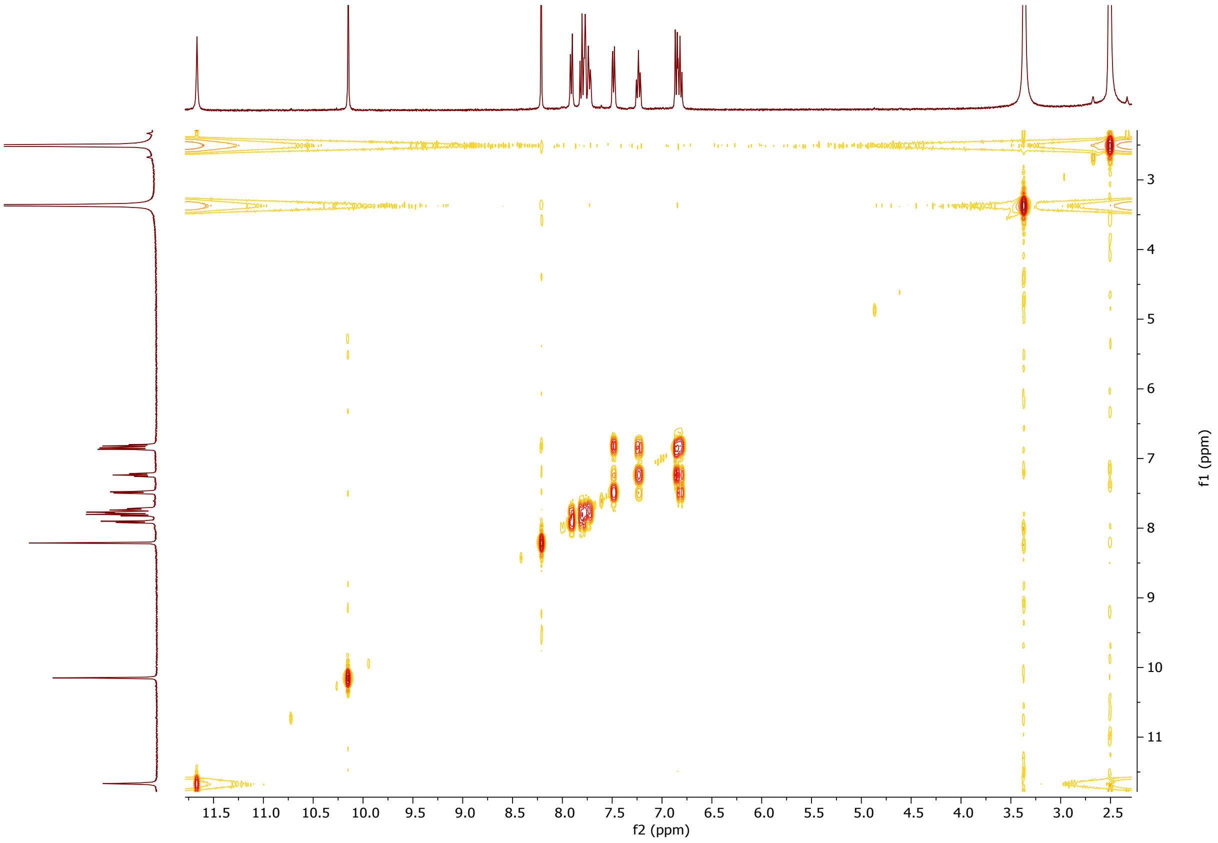


**Fig. S20.** COSY NMR spectrum of compound **6**

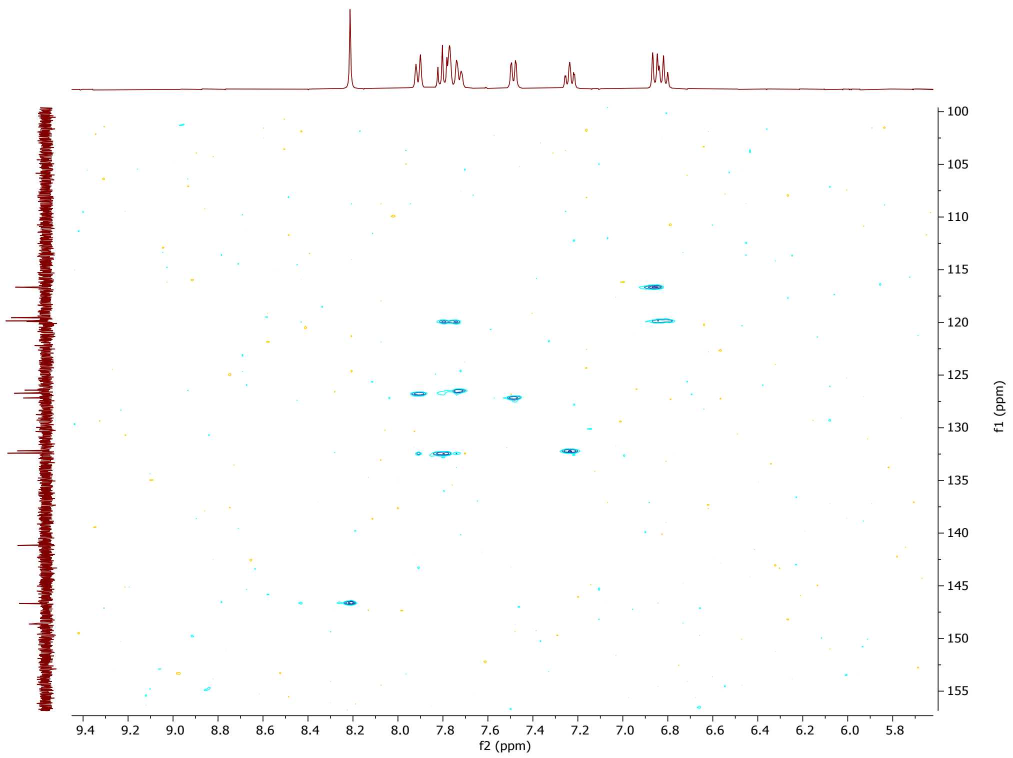


**Fig. S21.** HSQC NMR spectrum of compound **6**

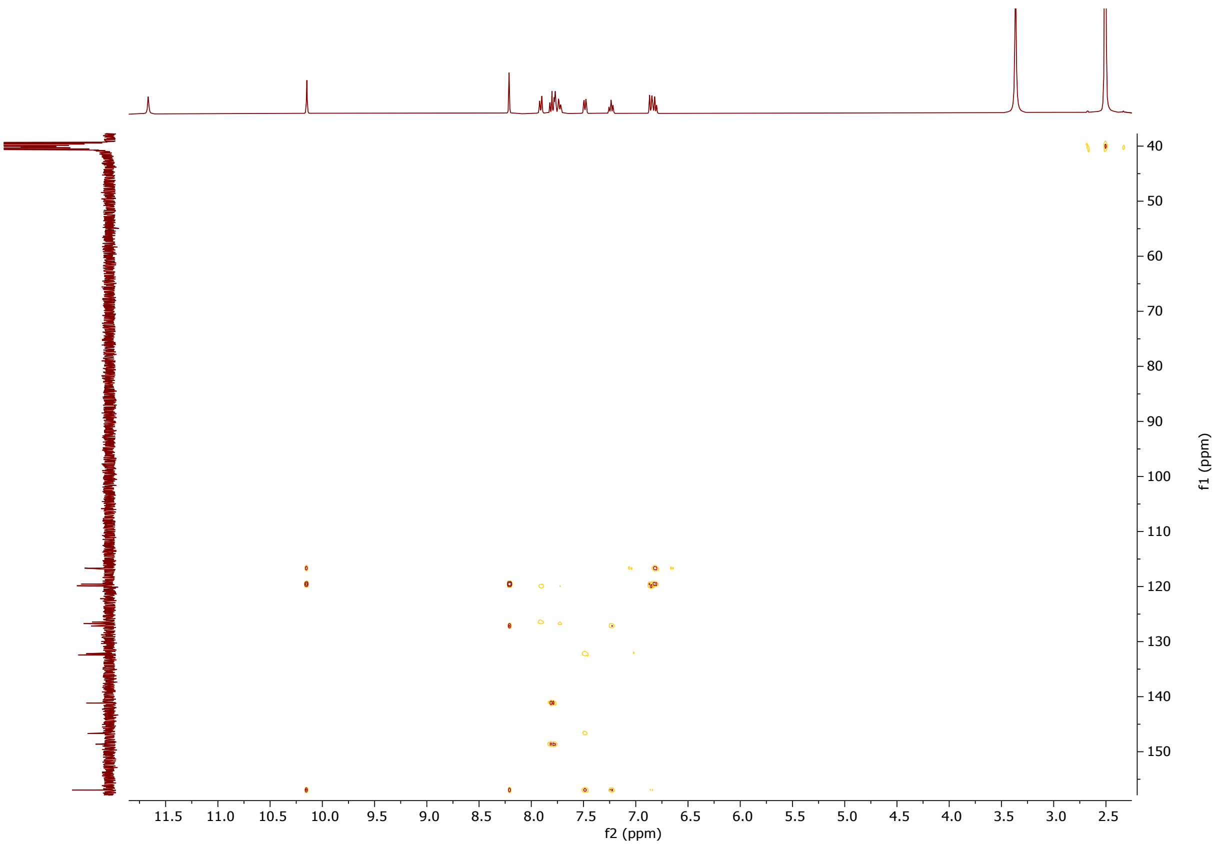

**Fig. S22.** HMBC NMR spectrum of compound **6**

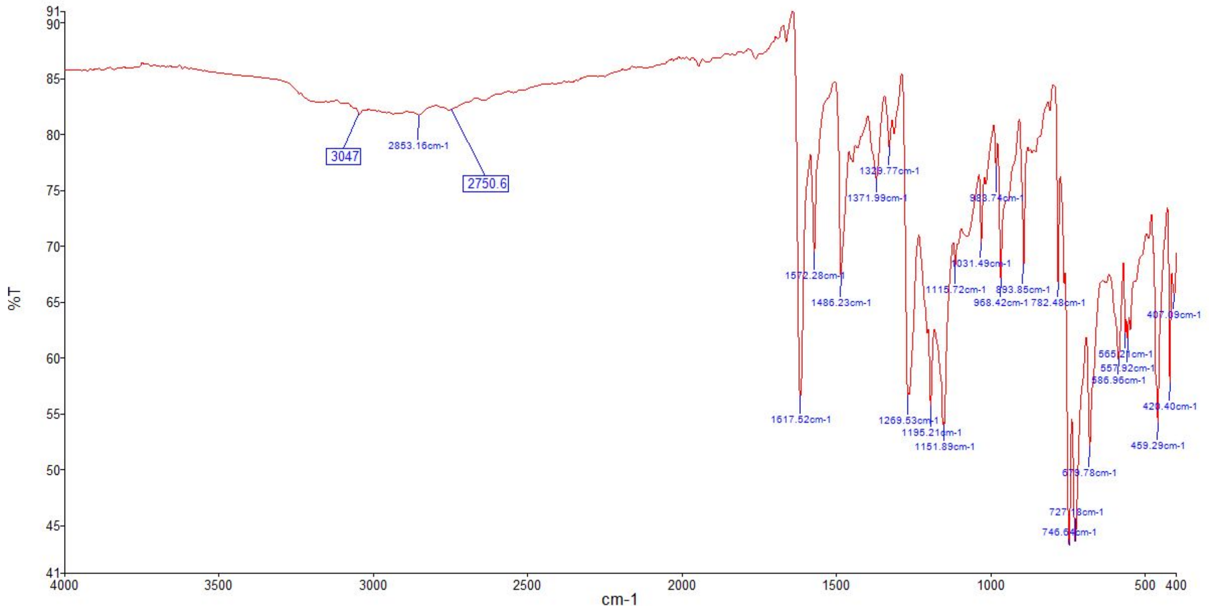


**Fig. S23**. FTIR spectrum of compound **7**

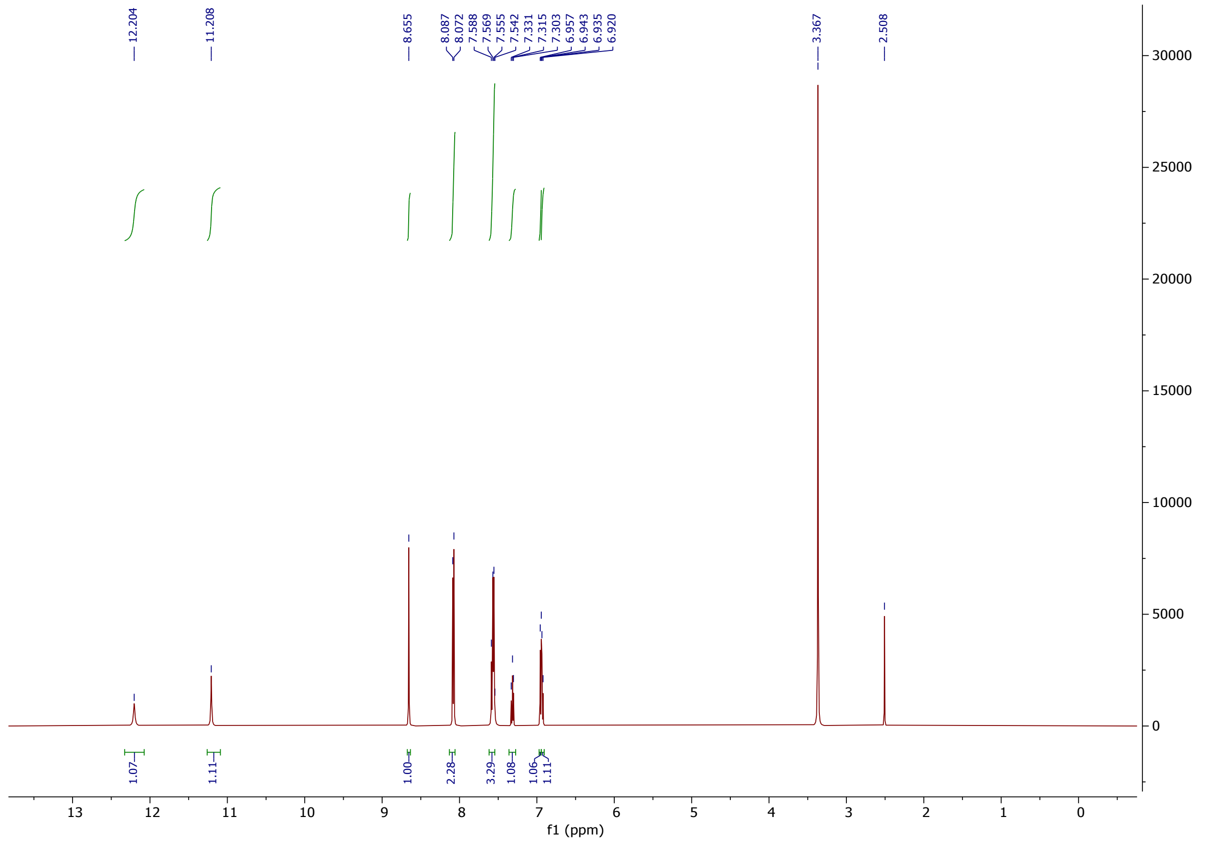

**Fig. S24.** $^{1}H$ NMR spectrum of compound **7**

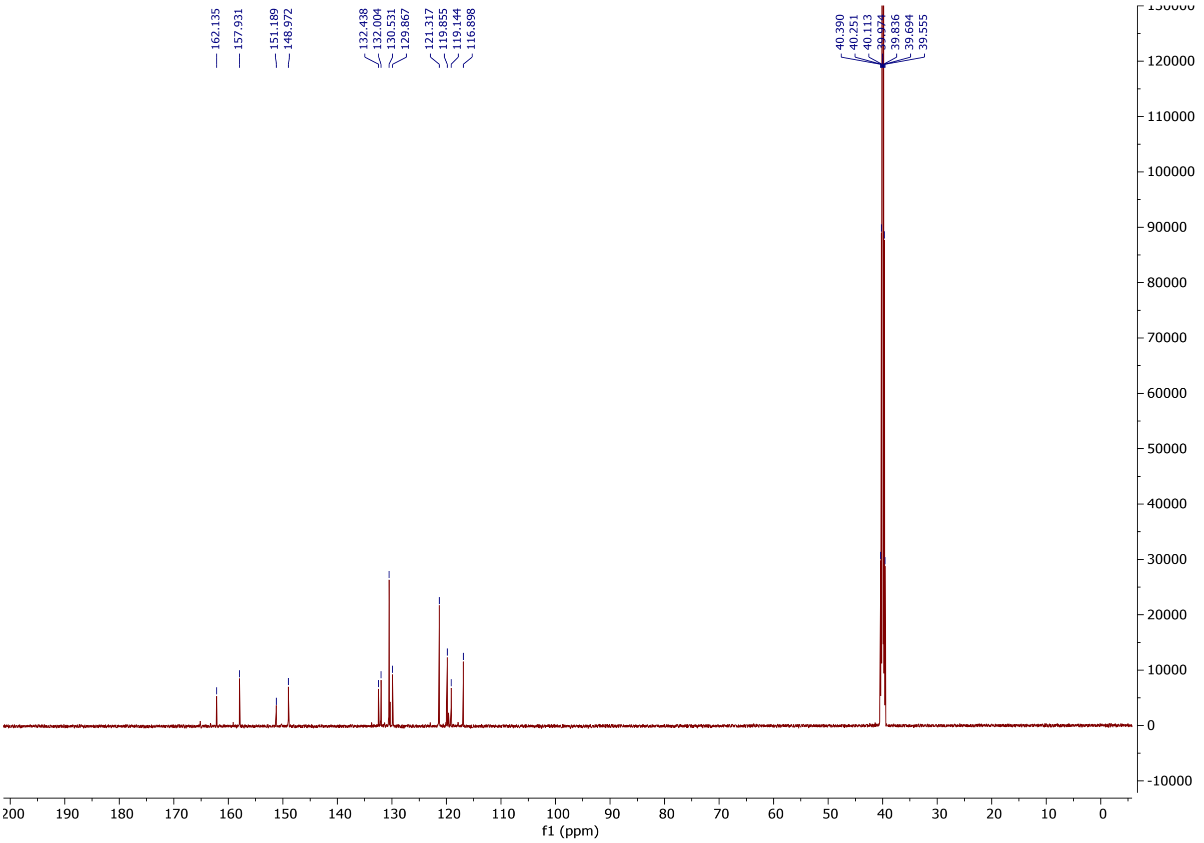


**Fig. S25.** $^{13}C$ NMR spectrum of compound **7**

## Table S1. Compound 1-7 water solubility

| Compound | Solubility | |
|---|---|---|
| 1 | Water Solubility | |
| | Log *S* (ESOL) | -3.27 |
| | Solubility | 1.59e-01 mg/ml ; 5.39e-04 mol/l |
| | Class | Soluble |
| | Log *S* (Ali) | -3.73 |
| | Solubility | 5.53e-02 mg/ml ; 1.88e-04 mol/l |
| | Class | Soluble |
| | Log *S* (SILICOS-IT) | -4.68 |
| | Solubility | 6.13e-03 mg/ml ; 2.08e-05 mol/l |
| | Class | Moderately soluble |
| 2 | Water Solubility | |

| | | |
|---|---|---|
| | Log *S* (ESOL) | -3.95 |
| | Solubility | 3.84e-02 mg/ml ; 1.12e-04 mol/l |
| | Class | Soluble |
| | Log *S* (Ali) | -4.55 |
| | Solubility | 9.79e-03 mg/ml ; 2.84e-05 mol/l |
| | Class | Moderately soluble |
| | Log *S* (SILICOS-IT) | -5.26 |
| | Solubility | 1.89e-03 mg/ml ; 5.50e-06 mol/l |
| | Class | Moderately soluble |
| 3 | Water Solubility | |
| | Log *S* (ESOL) | -3.95 |
| | Solubility | 3.84e-02 mg/ml ; 1.12e-04 mol/l |
| | Class | Soluble |
| | Log *S* (Ali) | -4.55 |
| | Solubility | 9.79e-03 mg/ml ; 2.84e-05 mol/l |
| | Class | Moderately soluble |
| | Log *S* (SILICOS-IT) | -5.26 |
| | Solubility | 1.89e-03 mg/ml ; 5.50e-06 mol/l |
| | Class | Moderately soluble |
| 4 | Water Solubility | |
| | Log *S* (ESOL) | -3.95 |
| | Solubility | 3.84e-02 mg/ml ; 1.12e-04 mol/l |
| | Class | Soluble |
| | Log *S* (Ali) | -4.55 |
| | Solubility | 9.79e-03 mg/ml ; 2.84e-05 mol/l |
| | Class | Moderately soluble |
| | Log *S* (SILICOS-IT) | -5.26 |
| | Solubility | 1.89e-03 mg/ml ; 5.50e-06 mol/l |
| | Class | Moderately soluble |
| 5 | Water Solubility | |
| | Log *S* (ESOL) | -4.16 |
| | Solubility | 2.50e-02 mg/ml ; 6.94e-05 mol/l |

| | | |
|---|---|---|
| | Class | Moderately soluble |
| | Log *S* (Ali) | -5.05 |
| | Solubility | 3.20e-03 mg/ml ; 8.89e-06 mol/l |
| | Class | Moderately soluble |
| | Log *S* (SILICOS-IT) | -4.99 |
| | Solubility | 3.69e-03 mg/ml ; 1.02e-05 mol/l |
| | Class | Moderately soluble |
| 6 | Water Solubility | |
| | Log *S* (ESOL) | -4.16 |
| | Solubility | 2.50e-02 mg/ml ; 6.94e-05 mol/l |
| | Class | Moderately soluble |
| | Log *S* (Ali) | -5.05 |
| | Solubility | 3.20e-03 mg/ml ; 8.89e-06 mol/l |
| | Class | Moderately soluble |
| | Log *S* (SILICOS-IT) | -4.99 |
| | Solubility | 3.69e-03 mg/ml ; 1.02e-05 mol/l |
| | Class | Moderately soluble |
| 7 | , Water Solubility | |
| | Log *S* (ESOL) | -4.16 |
| | Solubility | 2.50e-02 mg/ml ; 6.94e-05 mol/l |
| | Class | Moderately soluble |
| | Log *S* (Ali) | -5.05 |
| | Solubility | 3.20e-03 mg/ml ; 8.89e-06 mol/l |
| | Class | Moderately soluble |
| | Log *S* (SILICOS-IT) | -4.99 |
| | Solubility | 3.69e-03 mg/ml ; 1.02e-05 mol/l |
| | Class | Moderately soluble |
